# Two diverse carriers are better than one: A case study in α‐particle therapy for prostate specific membrane antigen‐expressing prostate cancers

**DOI:** 10.1002/btm2.10266

**Published:** 2021-11-17

**Authors:** Dominick Salerno, Alaina Howe, Omkar Bhatavdekar, Anders Josefsson, Jesus Pacheco‐Torres, Zaver M. Bhujwalla, Kathleen L. Gabrielson, Stavroula Sofou

**Affiliations:** ^1^ Chemical and Biomolecular Engineering (ChemBE) Institute for NanoBioTechnology (INBT) Johns Hopkins University Baltimore Maryland USA; ^2^ Russell H. Morgan Department of Radiology and Radiological Science Johns Hopkins University Baltimore Maryland USA; ^3^ Molecular and Comparative Pathobiology Johns Hopkins University Baltimore Maryland USA; ^4^ Sidney Kimmel Comprehensive Cancer Center, Cancer Invasion & Metastasis Program, Department of Oncology Johns Hopkins University Baltimore Maryland USA

**Keywords:** Actinium‐225, alpha‐particles, antibody, liposomes, prostate cancer, PSMA, tumor microdistributions

## Abstract

Partial and/or heterogeneous irradiation of established (i.e., large, vascularized) tumors by α‐particles that exhibit only a 4–5 cell‐diameter range in tissue, limits the therapeutic effect, since regions not being hit by the high energy α‐particles are likely not to be killed. This study aims to mechanistically understand a delivery strategy to uniformly distribute α‐particles within established solid tumors by simultaneously delivering the same α‐particle emitter by two diverse carriers, each killing a different region of the tumor: (1) the cancer‐agnostic, but also tumor‐responsive, liposomes engineered to best irradiate tumor regions far from the vasculature, and (2) a separately administered, antibody, targeting any cancer‐cell's surface marker, to best irradiate the tumor perivascular regions. We demonstrate that on a prostate specific membrane antigen (PSMA)‐expressing prostate cancer xenograft mouse model, for the same total injected radioactivity of the α‐particle emitter Actinium‐225, any radioactivity split ratio between the two carriers resulted in better tumor growth inhibition compared to the tumor inhibition when the total radioactivity was delivered by any of the two carriers alone. This finding was due to more uniform tumor irradiation for the same total injected radioactivity. The killing efficacy was improved even though the tumor‐absorbed dose delivered by the combined carriers was lower than the tumor‐absorbed dose delivered by the antibody alone. Studies on spheroids with different receptor‐expression, used as surrogates of the tumors' avascular regions, demonstrated that our delivery strategy is valid even for as low as 1+ (ImmunoHistoChemistry score) PSMA‐levels. The findings presented herein may hold clinical promise for those established tumors not being effectively eradicated by current α‐particle radiotherapies.

## 1 INTRODUCTION

Metastatic castration‐resistant prostate cancer (CRPC) is lethal and incurable, leading to the death of 31,000 men in the United States annually. Expression of the prostate specific membrane antigen (PSMA) is conserved in several of these advanced tumors.^1^, ^2^, ^3^ Currently, there is tremendous clinical promise for PSMA‐targeted alpha‐particle (α‐particle) therapies against soft‐tissue metastases of prostate cancer^4^, ^5^ for patients who were resistant to or ineligible for other therapies. However, the success of these α‐particle approaches against soft‐tissue metastases is expected to be most successful against relatively early disease.^4^, ^6^, ^7^

Alpha‐particles are high energy, short‐range particles (traveling in tissue up to 4–5 cell diameters). They physically break DNA molecules (causing double‐strand breaks—the most difficult to repair type of DNA damage) as they traverse the cell nucleus. The complexity and level of rapidly induced DNA damage overwhelms cellular repair mechanisms.^8^, ^9^ The inability to repair this type of DNA damage is the reason that α‐particles are impervious to most cancer cell resistance mechanisms,^10^, ^11^ if optimally delivered. However, the short‐range of α‐particles in tissue that enables localized irradiation with minimal toxicities to the surrounding healthy sites, also limits uniform irradiation of large tumors; this is because it is coupled with the diffusion‐limited penetration depths of traditional radionuclide carriers resulting, therefore, in partial tumor irradiation, potentially limiting efficacy.

We have recently discovered that we can improve the uniformity of distributions of α‐particles within established tumors when the same total radioactivity is equally split between two separate and diverse types of carriers, each preferentially killing a different region of the tumor^12^: (1) the tumor‐responsive liposomes that upon tumor uptake release in the interstitium a highly diffusing form of their radioactive payload (^225^Ac‐DOTA), which then penetrates the deeper parts of tumors where antibodies do not reach, and (2) a separately administered, less‐penetrating radiolabeled‐antibody, irradiating the tumor perivascular regions from where the liposomes' contents clear too fast. Our tumor‐responsive liposomes are composed of membranes forming phase‐separated lipid domains (resembling lipid patches) with lowering pH.^13^ During circulation in the blood, such liposomes comprise well‐mixed, uniform membranes that stably retain their encapsulated contents. In the acidic tumor interstitium (pH_e_ ~6.7–6.5^14^) lipid‐phase separation results in formation of lipid patches that span the bilayer, creating transient lipid‐packing defects along the patch boundaries, and enabling release of encapsulated agents. The liposomes also have an adhesive property that enables them to bind to the tumors' extracellular matrix, delaying their clearance from tumors.^15^, ^16^ On a proof‐of‐concept human epidermal growth factor receptor 2‐positive breast cancer mouse model, the equal split of injected radioactivity between the two separate carriers improved the tumor growth inhibition compared to the tumor inhibition observed at the same total injected radioactivity when treated by any of the carriers alone.

In the present study, (1) we investigate how the *different expression levels of the targeted receptor PSMA* on prostate cancers may affect the microdistributions of the PSMA‐targeting antibody and, therefore, the best radioactivity split ratio(s) for inhibiting cancer cell growth in the presence of transport barriers, and (2) we systematically interrogate the effect of *different radioactivity split ratios between the two carriers* (tumor‐responsive liposomes and the PSMA‐targeting antibody) on inhibiting the growth of PSMA‐expressing prostate cancers in vivo.

In particular, this study was motivated by the finding that PSMA expression in prostate cancer has been reported to exhibit variability and heterogeneity.^17^ On multicellular spheroids of variable size that were utilized as surrogates of the avascular regions within solid tumors, formed by prostate cancer cells with different expression levels of PSMA and different intrinsic sensitivities to Actinium‐225 (^225^Ac), we explored the killing efficacy of α‐particle radiotherapy delivered by several different radioactivity split ratios between the two carriers. On mice bearing PSMA‐positive prostate cancer xenografts, the biodistributions/dosimetry, tumor growth inhibition, and toxicities were evaluated for the same total injected radioactivity that was delivered by different split ratios between the two carriers. Our findings demonstrate that our delivery strategy is, in principle, applicable to cells with different levels of expression of the targeted surface marker, and it is, in a sense, tumor agnostic, since only the targeting antibody needs to be specific to the cell surface marker.

## 2 RESULTS

### 2.1 Carrier characterization

Table 1 shows that, under acidic conditions, both tumor‐responsive properties of liposomes were activated: (1) the zeta potential of liposomes that was previously shown to promote adhesion to the tumor's extracellular matrix, became more positive (mostly due to protonation of the dimethyl ammonium propane (DAP) moiety^18^), and (2) the release of encapsulated contents was increased (due to the formation of separated lipid‐phases with defective boundaries^13^). The PSMA‐targeting antibody (Table 2) maintained its immunoreactivity after radiolabeling and retained the radioactivity after incubation in media for at least 24 h. The antibody exhibited approximately 50% internalization when incubated with PSMA‐expressing PC3‐PIP cells (Figure S1). For both carriers, the retention (and/or release) properties between ^225^Ac and Indium‐111 (^111^In) loaded carriers were comparable, validating the use of ^111^In as a surrogate for ^225^Ac in the K_D_ and biodistribution studies.^16^, ^19^

**TABLE 1:** Characterization of the tumor‐responsive liposomes loaded with ^225^Ac‐DOTA and/or with ^111^In‐DTPA

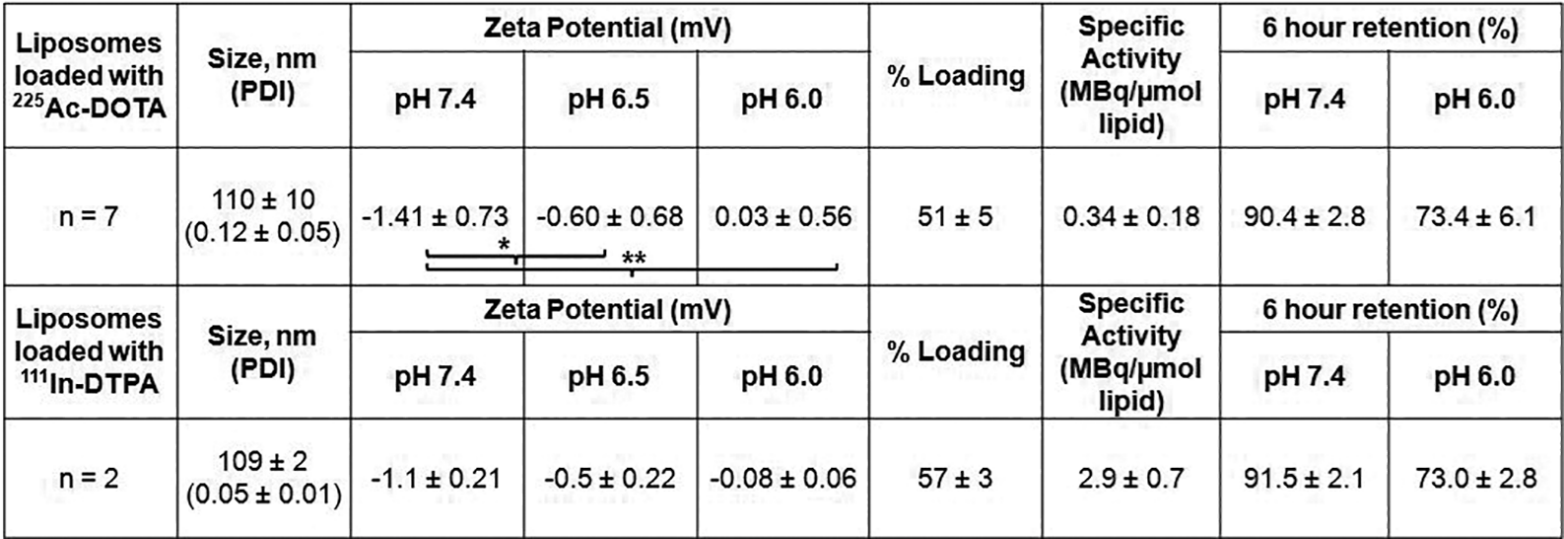

*Note*: *p < 0.05, **p < 0.01. Errors correspond to the standard deviation of *n* independent measurements (as indicated).

**TABLE 2:** Characterization of the PSMA‐targeting radiolabeled antibody (^225^Ac‐DOTA‐SCN‐antibody and ^111^In‐DTPA‐SCN‐antibody)

^225^Ac‐DOTA‐SCN‐Ab	Radiolabeling efficiency %	Immunoreactivity %	Specific activity (MBq/mgAb)	Radiochemical purity %	24 h retention % in media
*n* = 5	48 ± 10	74 ± 6	1.87 ± 0.73	96.6 ± 1.1	91.6 ± 2.7

^111^In‐DTPA‐SCN‐Ab	Radiolabeling efficiency %	Immunoreactivity %	Specific activity (MBq/mgAb)	Radiochemical purity %	24 h retention % in media
*n* = 2	53 ± 13	73 ± 5	152 ± 39	95.5 ± 2.1	92.5 ± 2.1

*Note*: Errors correspond to the standard deviation of *n* independent measurements (as indicated).

Abbreviation: PSMA, prostate specific membrane antigen.

### 2.2 Cell line characterization and survival assay

The expression levels of PSMA on C4‐2B, LNCaP, and PC3‐PIP prostate cancer cells were measured, using the PSMA‐targeting ^111^In‐DTPA‐SCN‐antibody, to be 126,000 ± 12,000, 210,000 ± 12,000, and 3,400,000 ± 78,000 copies per cell, respectively; the PSMA‐targeting antibody exhibited comparable K_D_ across all cell lines (Table S1 and Figure S2). These cell lines were chosen as representatives for low, moderate, and high levels of PSMA expression. The PC3 cell line did not express any measurable levels of PSMA, in agreement with previous reports.^20^ Each cell line formed 3D spheroids, which developed acidity in the interstitium (Figure S3), serving as an in vitro model of the avascular regions of solid tumors.

**FIGURE 1:**
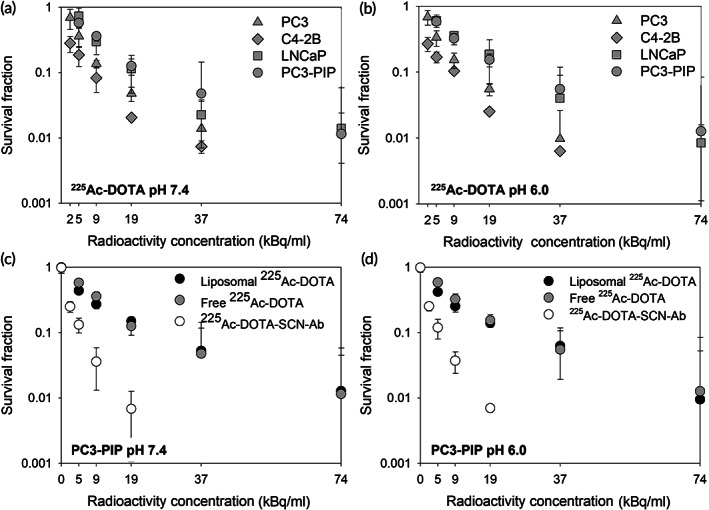
Colony survival of prostate cancer cell lines after a 6‐h incubation with free ^225^Ac‐DOTA at (a) pH 7.4 and (b) pH 6.0 (chosen to represent the lowest pH_e_ value in the tumor interstitium), and colony survival of the PC3‐PIP prostate cancer cell line after a 6‐h incubation with the tumor‐responsive liposomes loaded with ^225^Ac‐DOTA, and the PSMA‐targeting ^225^Ac‐DOTA‐SCN‐antibody at (c) pH 7.4 and (d) pH 6.0 (30 μg/ml total antibody concentration, corresponding to 100 times excess of prostate specific membrane antigen [PSMA] receptors used in the spheroid studies). Error bars correspond to the standard deviation of repeated measurements (*n* = 3–6 samples per condition, *n* = 2–4 independent runs)

On cell monolayers, in the absence of transport barriers, the killing efficacy of free ^225^Ac‐1,4,7,10‐tetraazacyclododecane‐1,4,7,10‐tetraacetic acid (DOTA) was independent of the extracellular pH for all cell lines (Figure 1a,b). The killing effect of free ^225^Ac‐DOTA and of ^225^Ac‐DOTA encapsulated in tumor‐responsive liposomes was the same for PC3‐PIP cells (Figure 1c,d), and for the other cell lines (Figure S4). This finding was expected since neither free ^225^Ac‐DOTA nor the liposomes preferentially associate with cells. Finally, the PSMA‐targeting ^225^Ac‐DOTA‐SCN‐antibody exhibited increasing killing efficacies with increasing expression of PSMA by cells (Figure 1a,b, compared to Figure S4).

### 2.3 Spheroid growth control

**FIGURE 2:**
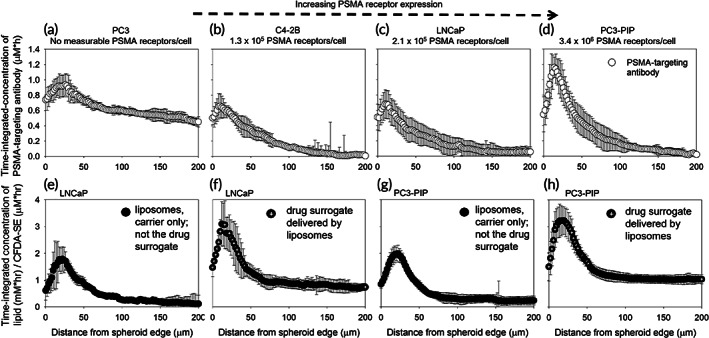
Time‐integrated microdistributions of the fluorescein isothiocyanate (FITC)‐labeled prostate specific membrane antigen (PSMA)‐targeting antibody (a through d: open symbols), DPPE‐rhodamine‐labeled liposomes (e, g: filled symbols), and CFDA‐SE used as the drug surrogate delivered by the liposomes (f, h: crossed symbols) on 400 μm in diameter prostate cancer spheroids with different expression levels of the targeted receptor; (e) and (g) show the time‐integrated microdistributions of the liposomes; in other words, of the carriers only and not their water‐soluble contents; (f) and (h) show the time‐integrated microdistributions of a (water soluble) drug surrogate that was delivered by the liposomes. The spatial distributions obtained at different timepoints (during carrier uptake by and clearance from spheroids) were integrated using the trapezoid rule along the spheroid radius. Error bars correspond to standard deviations of measurements on *n* = 3 equatorial spheroid sections per time point. Immunoreactivity of the fluorescently labeled antibody was: 73% ± 5%

Spheroids were utilized in this study as surrogates of the avascular regions of solid tumors. We show that, due to diffusion‐limited transport in the spheroid interstitium, the time‐integrated microdistributions of the specific antibody (Figure 2a–d) and of the liposomes (Figure 2e,g) exhibited accumulation that was limited to the first, approximately, 60 μm from the spheroid edge; liposomes were not detectable at the spheroid core. Antibodies were only detectable at the core of spheroids that did not express the targeting receptor PSMA (Figure 2a), indicative of the binding site barrier effect.^21^ As expected, increasing expression of the targeted receptor by cancer cells (direction from left to right) resulted in greater uptake of the antibody (both surface‐bound and internalized) by the cells closer to the spheroid edge. In contrast to this, the liposomes' released contents exhibited significant penetration reaching the spheroid center (Figure 2f,h); but only low retention in the first 10–15 μm from the spheroid edge, due to their fast clearance into the surrounding media. The spatial profiles of carriers per time point are shown in Figures S5 and S6.

**FIGURE 3:**
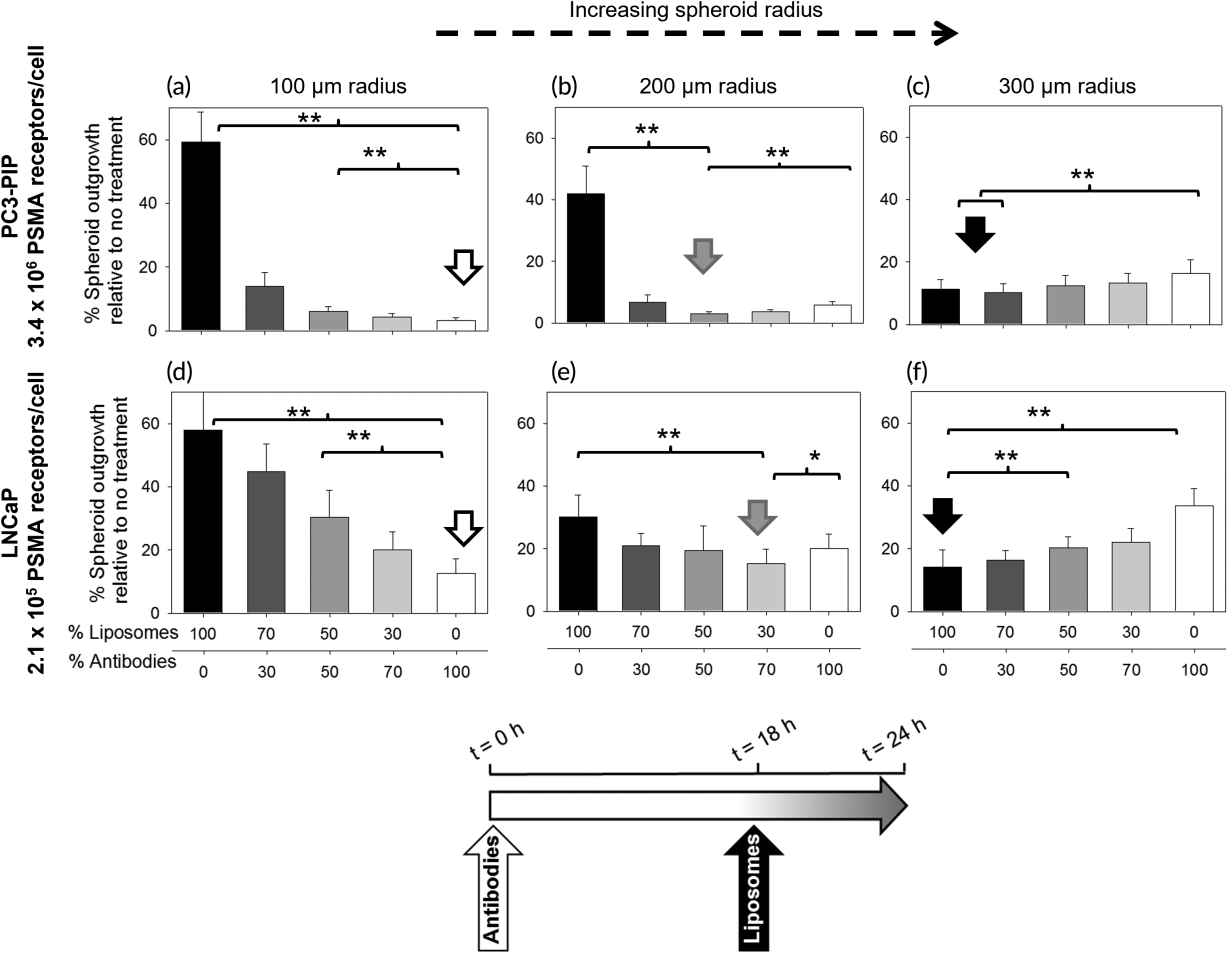
Best outgrowth inhibition of spheroids with increasing size, at same total radioactivity, was exhibited by radioactivity split ratios that favored greater liposome fractions. Extent of outgrowth inhibition of spheroids formed of PC3‐PIP cells (with high prostate specific membrane antigen [PSMA] expression, a–c) and of LNCaP cells (moderate PSMA expression, d–f) with varying sizes after treatment with different radioactivity split ratios between the tumor‐responsive liposomes loaded with ^225^Ac‐DOTA (liposomes, incubated for 6 h) and the PSMA‐targeting ^225^Ac‐DOTA‐SCN‐antibody (antibodies, incubated for 24 h, to approximately match the relative blood clearance kinetics of the carriers). The total radioactivity concentration was kept constant per spheroid size (a) 4.5 kBq/ml, (b) 9 kBq/ml, (c) 20 kBq/ml, (d) 4.5 kBq/ml, (e) 9 kBq/ml, (f) 13 kBq/ml. Error bars correspond to standard deviations of repeated measurements (*n* = 6 spheroids per condition, *n* = 3 independent liposome and antibody preparations). * indicates *p* < 0.05, **p < 0.01

In agreement with the above complementary microdistributions are the findings in Figure 3, where the greatest inhibition of spheroid growth (indicated by the vertical arrow) was exhibited: (1) by the radiolabeled antibody in the case of small spheroids (Figure 3a,d) with radius r = 100 μm, which is comparable to the 80–100 μm range of α‐particles in tissue, (2) by the radiolabeled tumor‐responsive liposomes in the case of large spheroids (Figure 3c,f) with radius r = 300 μm, which is three times the range of α‐particles, and (3) by a combination of the two carriers (Figure 3b,e) for spheroids with radius r = 200 μm, which is an intermediate value between the “small” and “large” spheroids, and is also considered a characteristic length in vivo beyond which vascularization is usually observed.^22^ These findings were independent of the expression levels of PSMA by cells (high levels on PC3‐PIP cells vs. moderate levels on LNCaP cells).

**FIGURE 4:**
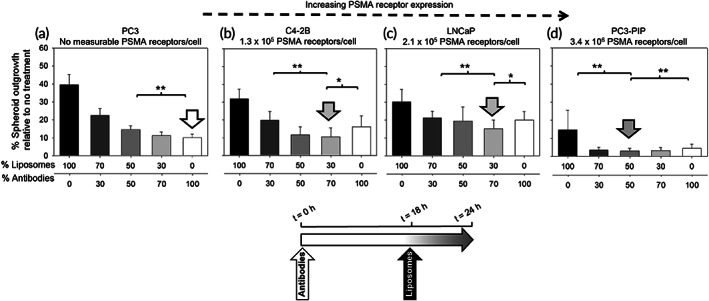
Best outgrowth inhibition of (same size) spheroids formed by cells with increasing prostate specific membrane antigen (PSMA)‐expression levels, at same total radioactivity, was exhibited by radioactivity split ratios that favored greater liposome fractions. Extent of outgrowth inhibition of spheroids (200 μm radius) formed by prostate cancer cells (with different PSMA copies per cell): (a) PC3 (PSMA not detected), (b) C4‐2B (1.3 × 10^5^), (c) LNCaP (2.1 × 10^5^), and (d) PC3‐PIP (3.4 × 10^6^) after treatment with different radioactivity split ratios between the tumor‐responsive liposomes loaded with ^225^Ac‐DOTA (liposomes, incubated for 6 h) and the PSMA‐targeting ^225^Ac‐DOTA‐SCN‐antibody (antibodies, incubated for 24 h). The total radioactivity concentration was kept constant across all spheroid types at 9 kBq/ml (a–d). Error bars correspond to standard deviations of repeated measurements (*n* = 6 spheroids per condition, *n* = 3 independent liposome and antibody preparations). * indicates *p* < 0.05, **p < 0.01

In addition, Figure 4 shows that on the same (intermediate) size spheroids (r = 200 μm) with increasing PSMA‐expression levels (left‐to‐right), the best inhibition of spheroid growth was exhibited when increasing fractions of the radioactivity were delivered by the liposomes. This is because the radiolabeled antibody delivers its therapeutic cargo mostly in the periphery of the spheroid (the so‐called binding site barrier effect)^12^, ^21^: increasing PSMA‐expression levels by cells required a lower fraction of the antibody‐delivered radioactivity, since the same levels of delivered radioactivity per cell could be reached at lower antibody concentrations given the increased numbers of receptors per cell. In Figure 4a, on spheroids that do not express detectable PSMA levels, both the liposomes and the antibodies behaved as nontargeting particles with the latter being significantly smaller. Note that each of the carriers was incubated with spheroids for different times: 24 h with the antibodies and 6 h with the liposomes, to approximately match their blood circulation half‐lives in mice. The (smaller) antibodies had, therefore, longer available time to penetrate deeper in these PSMA‐negative spheroids. The cold liposomes and the cold antibody did not affect the growth of any of the spheroids compared to the nontreated condition (Figure S7).

### 2.4 In vivo assessment

**FIGURE 5:**
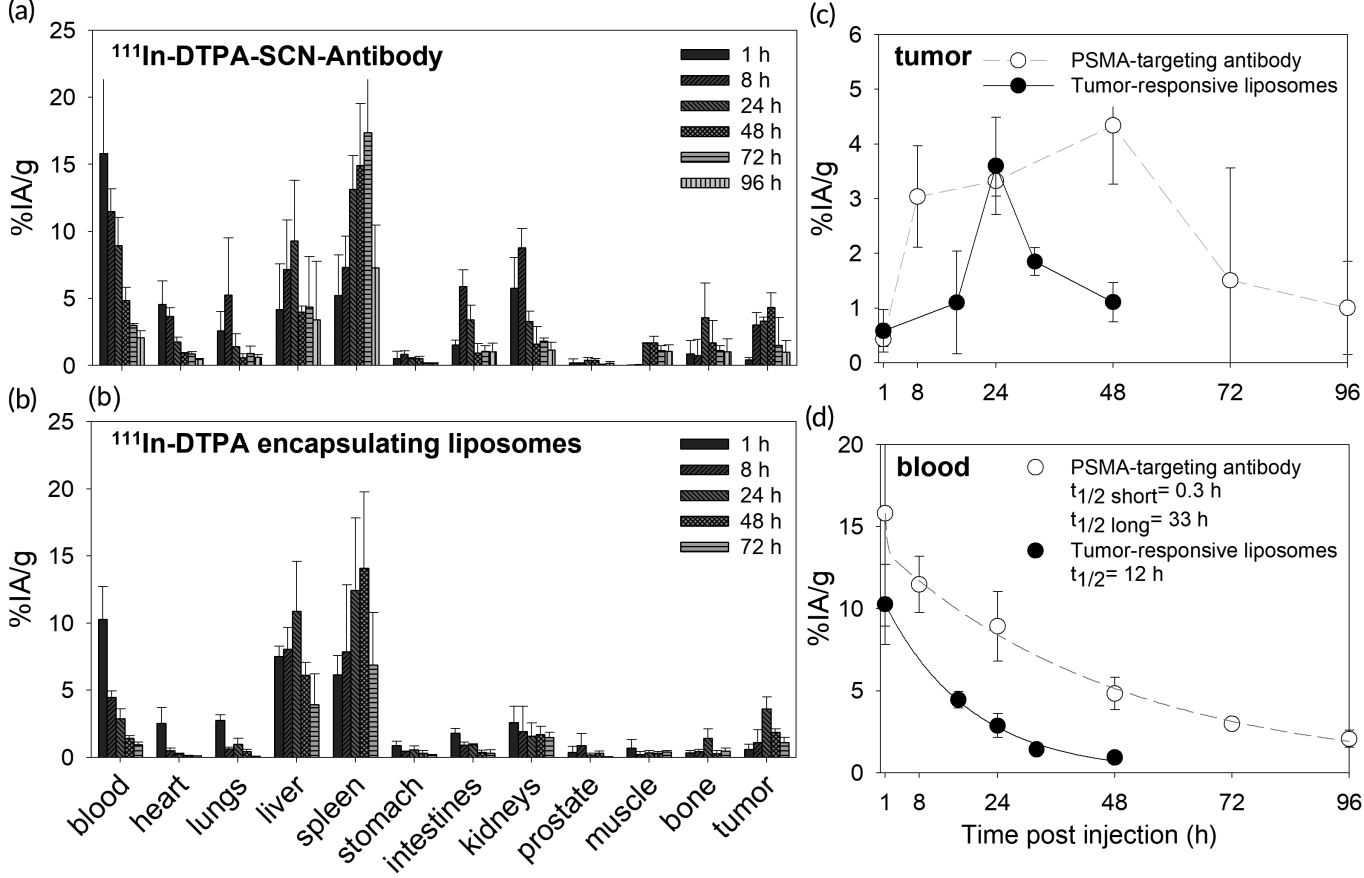
Biodistributions of (a) the prostate specific membrane antigen (PSMA)‐targeting ^111^In‐DTPA‐SCN‐antibody and the (b) tumor‐responsive liposomes loaded with ^111^In‐DTPA on NSG mice bearing PSMA‐overexpressing PC3‐PIP subcutaneous tumors. ^111^In was used as surrogate of the parent ^225^Ac. Comparison of the tumor uptake and clearance (c) and the blood clearance kinetics (d) of the two carriers. Error bars correspond to standard deviations between *n* = 3 mice per condition per time point

As previously reported, major off‐target sites for both carriers were the liver and spleen, as well as the kidneys for the PSMA‐targeting antibody (Table S2, Figure S8). Tumor uptake of the PSMA‐targeting ^111^In‐DTPA‐SCN‐antibody was significantly greater than that of ^111^In‐DTPA‐loaded liposomes (Figure 5c); this was partly attributed to the different blood clearance half‐lives of each carrier (Figure 5d). Table 3 shows the significant difference between the tumor‐absorbed doses when the same total radioactivity was injected in mice and was delivered by each carrier alone and/or by both carriers at equal ratios of injected radioactivity.

**TABLE 3:** Dosimetry results

	Absorbed dose (Gy)^a^					
Tissue	Antibody only		Liposomes only		Antibody:liposomes 50:50 (same total injected activity)	
	D	SD	D	SD	D	SD
Kidneys	0.46	0.04	0.32	0.14	0.39	0.07
Liver	1.47	6.73	0.51	0.10	0.99	3.37
Lung	0.52	1.88	0.04	0.01	0.28	0.94
Spleen	1.83	0.26	0.75	0.21	1.29	0.17
Tumor	0.34	0.04	0.12	0.02	0.23	0.02

^a^ Mean dose (D) and standard deviation (SD), not adjusted by relative biological effectiveness.

**FIGURE 6:**
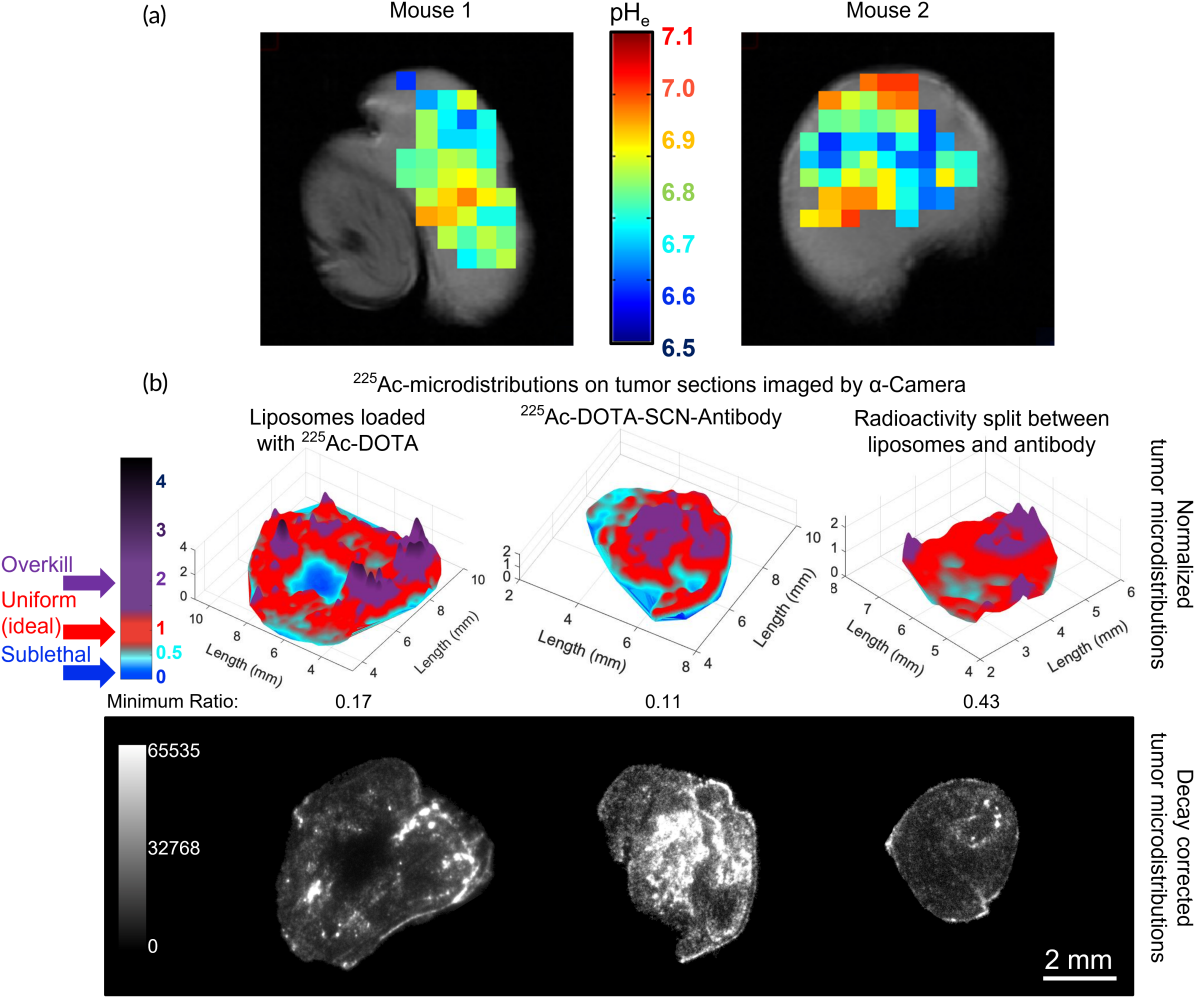
(a) Extracellular pH maps (pH_e_) of PC3‐PIP tumors acquired by MRSI. Tumor‐bearing mice were injected with 0.2 ml of the pH reporter ISUCA, and its measured chemical shift was then mapped into a pH_e_ value using the Henderson–Hasselbalch equation. Color‐coded pH_e_ values were overlaid onto anatomical MRI images encompassing the volume of interest. (b) Color maps of prostate specific membrane antigen (PSMA)‐positive PC3‐PIP prostate cancer tumor sections indicating deviation from uniformity (red) of delivered α‐particle radiotherapy. In all cases, mice were I.V. injected the same total radioactivity that was delivered by: (left) only carrier 1 (liposomes), (middle) only carrier 2 (PSMA‐targeting antibodies), (right) both carriers, and animals were sacrificed 24 h later. On top panel, shown are the pixel intensities of the α‐camera images of tumor sections that were normalized relative to the average intensity of the entire tumor section. Second panel shows the decay‐corrected α‐camera images. Red: indicates uniform/ideal radionuclide distributions; purple: areas where more than necessary radioactivity is delivered; blue: areas receiving too low radioactivities to result in complete cell kill

The extracellular pH_e_ maps of PSMA‐expressing PC3‐PIP subcutaneous tumors confirmed the development of acidity in the interstitium, with regions reaching as low pH_e_ values as 6.5 (Figure 6a); these pH_e_ values were adequate to activate both the release and adhesion properties on the tumor‐responsive liposomes (Table 1).^12^, ^16^

The α‐camera images of tumor sections on the top panel in Figure 6b show the pixel intensities of tumor sections that were normalized relative to the average intensity of the entire tumor section. At same total administered radioactivity, the images demonstrate more uniform microdistributions (red‐colored areas) of the delivered radioactivity when both carriers were utilized (Figure 6b, right side), compared to each carrier alone (Figure 6b, left side and middle). (Regions colored in red indicate pixel ratios close to unity; indicative of uniform microdistributions, where the local delivered radioactivity is close to the section average. Regions in purple [blue] indicate pixel ratios greater [lower] than unity potentially being correlated with local delivery of excessive [sublethal] radioactivities.)

**FIGURE 7:**
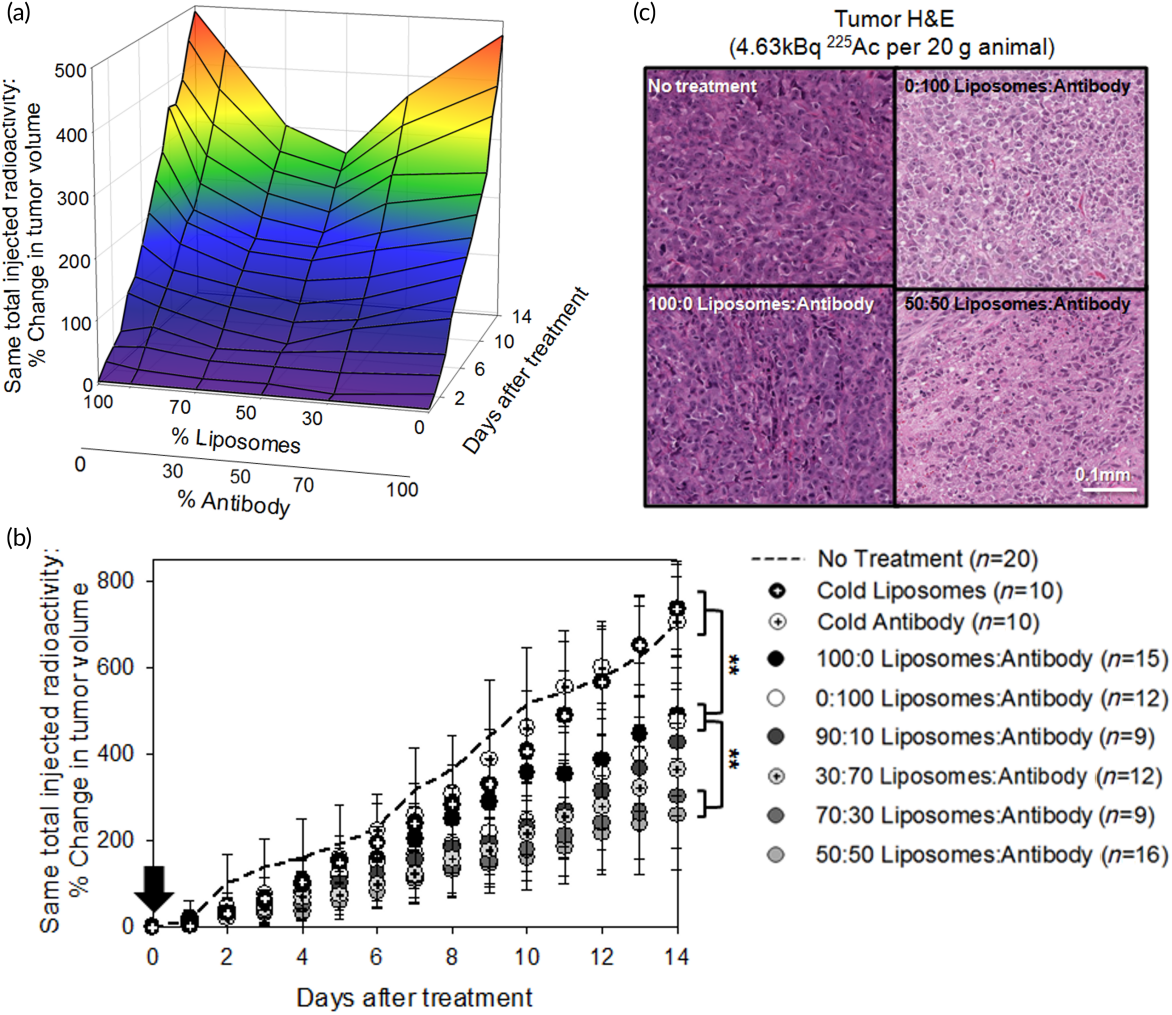
(a) 3D representation of the percent change in PC3‐PIP tumor volume as a function of time and the radioactivity split ratios between the two carriers. The nontreated animals and the effect of cold carriers are not included. (b) Percent change of the PC3‐PIP subcutaneous xenograft volume over time after a single I.V. injection of 4.63 kBq ^225^Ac (black vertical arrow), per 20 g NSG mouse, delivered by tumor‐responsive liposomes loaded with ^225^Ac‐DOTA (black symbols), or the prostate specific membrane antigen (PSMA)‐targeting ^225^Ac‐DOTA‐SCN‐antibody (white symbols) and/or different radioactivity split ratios between the two carriers (grayscale symbols) at same total administered radioactivity. No treatment (dashed line), the cold tumor‐responsive liposomes, and the cold PSMA‐targeting antibody (black/white symbols with cross sign) exhibited similar behavior. Percent change in tumor volume for each mouse on each day was calculated by (V_t_‐V_o_)/V_o_ × 100%. Data points are the mean values and error bars the standard deviations of n animals per group (as indicated in the legend). Significance was calculated with one‐way analysis of variance (*p*‐value < 0.05). * indicates 0.01 < *p*‐values<0.05; ** *p*‐values<0.01. (c) H&E stained tumor sections. Scale bar = 100 μm

For assessment of therapeutic efficacy, mice bearing subcutaneous PC3‐PIP xenografts were administered I.V. once 4.63 kBq in total that was delivered either by the PSMA‐targeting antibody alone, or by the tumor‐responsive liposomes alone, and/or by different radioactivity split ratios between the two carriers injected simultaneously, in addition to the cold liposomes and the cold antibody. Inhibition of tumor growth was better in all cases where both carriers were utilized to deliver the same total radioactivity, compared to either carrier individually delivering it (Figure 7a,b). The greatest tumor growth inhibition was observed with the 50:50 and 70:30 liposome:antibody radioactivity split ratios (Figure 7a,b, Figure S9, and Figure S10 for growth inhibition of individual tumors). Cold liposomes and cold antibody did not affect tumor growth.

Animal survival was not a meaningful endpoint for this animal model because of the aggressiveness of tumor progression. Nontreated PC3‐PIP tumors (Figure S11, top panel) exhibited large regions of necrotic tissue in accordance with the aggressive nature of this tumor model. However, in the cell‐dense viable tissue regions, increased collagen and fewer cells were consistently observed in the treatment conditions when compared to the no treatment (Figure 7c). Among the different treatment conditions, the tumor regions of animals that were treated with the same total radioactivity equally split between the two carriers, exhibited the least cell density.

Histopathology analysis of H&E stained organ sections showed no noteworthy toxicities in the heart, lungs, liver, or kidneys across all constructs at the time of animal sacrifice (14 days post‐treatment) (Figure S12). The spleen of mice treated with α‐particles showed low levels of extramedullary hematopoiesis (EMH), which is to be expected as the spleen was observed as a major site of both liposome and antibody uptake. As previously shown in maximum tolerated dose (MTD) studies with these tumor‐responsive liposomes (evaluated to be 5.55 kBq per 20 g NSG mouse),^16^ the reduced EMH is expected to reverse post‐treatment, and spleens were fully recovered after 9.5 months when tumor‐free mice were treated at this dose. Throughout the period of 14 days from treatment, all animal weights were maintained within 10% of the weight on the day of initiation of therapy (Figure S13).

Intravenous administration of 4.63 kBq per 20 g mouse of the PSMA‐targeting ^225^Ac‐DOTA‐SCN‐antibody did not result in deaths of tumor‐free mice. Pathology evaluation of these animals did not detect toxicities on animals euthanized 1 month after radioactivity was injected (Figure S14).

## 3 DISCUSSION

We have hypothesized and demonstrated that in order to improve the killing efficacy against established, soft‐tissue solid tumors, more uniform microdistributions of α‐particle emitters within the tumors are required, as opposed to just more α‐particle radioactivity without considering their microdistributions. This was shown to be due to the larger fraction of tumor cells becoming exposed to the cytotoxic agents, as opposed to only a smaller fraction of the tumor cells receiving greater doses. To enable this, we administered the same total α‐particle radiotherapy using combinations of separate carriers (tumor‐responsive liposomes and targeting antibodies) that resulted in complementary microdistributions of the α‐particle emitter ^225^Ac within established solid tumors.

In particular, we studied PSMA‐positive PC3‐PIP prostate cancer tumors in vivo, which proved to be particularly heterogeneous in their morphology (as shown in Figure S11), and we demonstrated that the same total α‐particle radioactivity delivered by any combinations of the two carriers (the liposomes and a PSMA‐targeting antibody) improved overall efficacy (i.e., tumor growth inhibition). While the best response at the same total injected radioactivity was seen when the radioactivity was split equally between the two carriers, all ratios of radioactivity split between the carriers resulted in better tumor growth inhibition than the inhibition achieved by the same total dose delivered by either carrier alone. These findings suggest that *any ratio* of carriers, chosen to split the same total injected radioactivity, results in more uniform α‐particle tumor microdistributions compared to the microdistributions enabled by any single carrier alone. Notably, the tumor‐absorbed dose delivered by any carrier combination was lower than the dose delivered by the antibody alone (as shown in Table 3). The best tumor growth inhibition was achieved when the same total radioactivity was equally split between the two carriers that resulted in 32% less tumor‐absorbed dose (0.23 vs. 0.34 Gy) compared to the tumor‐absorbed dose delivered by the antibody alone.

The improved killing efficacy of the same radioactivity when delivered by combinations of the two carriers was also observed in vitro on multicellular spheroids—used as surrogates of tumors' avascular regions—that were formed by prostate cancer cells with varying levels of PSMA expression. These studies suggest that our delivery strategy for α‐particle emitters can be effective even when the targeted marker expression is as low as 130,000 copies per cell (corresponding to roughly 1+ by ImmunoHistoChemistry score^23^).

From an engineering perspective, tumors, in vivo, may be considered collections of avascular cancer cell‐populated regions with variable characteristic lengths (or sizes) that could be represented by the different spheroid diameters. The distributions of these avascular distances may vary with the type and size of solid tumors and may also vary across established metastases of the same patient. Although detailed mathematical modeling, which we currently evaluate, could be very informative regarding the significance of each of the two complementary delivery mechanisms, namely of diffusion (of the released contents from liposomes) and of the binding/reaction (of the therapeutics stably conjugated on the antibodies), it is remarkable that, in practice, almost any split ratio of the total radioactivity between the two carriers improved the tumor growth inhibition compared to any carrier alone. This result may be indicative of the physiologic “balance” among avascular regions of “short,” “medium,” and “long” lengths. We envision a scenario of pretreatment scanning of patients with the aim to reconstruct the 3D tumor vascular networks, from which a mathematical model would predict the optimum radioactivity split ratio between the carriers for best tumor growth inhibition bound, of course, by the constraints of off‐target toxicities.

The characterization of the intratumoral distributions of delivered radioactivity refers to the microscopic scale, and only for tumor regions that exhibited comparable “topography” in terms of neovasculature densities. The macroscopic structure of the PC3‐PIP tumors was found, in this study, to be particularly variable and heterogeneous, probably due to the fast tumor growth; necrotic regions were found not only in the center of tumor sections but often also close to their macroscopic periphery (Figure S11). The macroscopic heterogeneities of tumors are, nevertheless, independent of our findings in spheroids; the latter were treated as surrogates of the tumor avascular regions. Therefore, they help us understand the spatiotemporal distributions and effects of different carriers at the microscopic scale, and are not expected to characterize the necrotic regions of tumors, or the distributions of agents at the macroscopic tumor level.

The efficacy studies were performed at combined administered radioactivities (4.63 kBq/20 g mouse) that did not cause measurable toxicities in tumor‐free mice when the same total radioactivity was delivered only by the antibody or by the liposomes alone.^16^ Potential dose‐limiting organs, in mice, were the liver and spleen that exhibited significant absorbed doses; kidney irradiation by the liposomes was previously shown not to be a concern.^16^

Dividing the same total radioactivity into both carriers did not significantly change the already substantial delivered dose to the liver and/or spleen, and only slightly decreased the dose to the kidneys (Table 3) compared to the dose delivered by the antibody alone. Regarding the liver and spleen irradiation in our studies, we have previously shown that, even at the MTD, hepatic and splenic toxicities were not observed 9.5 months after administration of radioactivity.^16^ In mice, renal toxicities caused by radiolabeled antibodies have been shown to be, in principle, addressable.^24^, ^25^ Potential interrogation of delivery scheduling (simultaneous injection of both carriers vs. sequential injection with a time gap), in addition to fractionation of the radioactivity delivered by each carrier, may minimize any toxicities. Interestingly, in humans, clinical trials with ^225^Ac‐labeled antibodies have not indicated the kidneys as the dose‐limiting organ^26^ although PSMA‐targeting smaller‐molecule vectors of ^225^Ac could be affected by the kidney delivered dose.^7^, ^27^

Considering requirements for potential translation to humans, the tumors' vascular permeability to liposomes and the acidification of the tumor microenvironment are critical for this strategy. It has been shown in clinical studies that, should the tumor vasculature have measurable permeability to liposomes, then liposomal carriers result in significant uptake in solid tumors, and this was shown to be strongly correlated with improved inhibition of tumor growth.^28^ In addition, acidity in the tumor interstitium, that is, necessary to trigger the tumor responsive properties of liposomes, has been observed in a variety of human prostate cancer tumors,^29^, ^30^ at levels comparable to those seen in the mouse model used herein. Tumor acidification usually correlates with aggressive tumors,^31^ for which this treatment strategy could be appropriate due to the intrinsic high cytotoxicity of the delivered α‐particles and the relatively low administered radioactivities required to elicit therapeutic effects without reaching toxic levels of absorbed doses at normal organs.

## 4 CONCLUSION

This study establishes the mechanistic underpinnings of a delivery strategy to enhance the killing efficacy of α‐particles against established, soft‐tissue solid tumors by improving the uniformity of their intratumoral microdistributions. The strategy employs two diverse carriers of the α‐particle emitter ^225^Ac, each primarily depositing their cytotoxic cargo at different areas of the same tumor: (1) tumor‐responsive liposomes are engineered to best irradiate tumor regions far from the vasculature, and, (2) a, separately administered, radiolabeled antibody to best irradiate the tumor perivascular regions. For prostate cancers, the antibody was chosen to target the PSMA surface marker on the tumor cells. With current clinical trials evaluating targeted ^225^Ac delivery to solid tumors, including the targeting of PSMA receptor, the findings presented herein may hold clinical promise for the established (i.e., large, vascularized) tumors not being effectively eradicated by current approaches.

## 5 MATERIALS AND METHODS

### 5.1 Materials

All lipids were purchased from Avanti Polar Lipids (Alabaster, AL, USA), including 1,2‐diarachidoyl‐sn‐glycero‐3‐phosphocholine (20PC), 1,2‐dipalmitoyl‐sn‐glycero‐3‐phospho‐l‐serine (sodium salt) (DPPS), and 1,2‐dipalmitoyl‐sn‐glycero‐3‐phosphoethanolamine‐N‐(lissamine rhodamine B sulfonyl) (ammonium salt) [1,2‐dipalmitoyl‐sn‐glycero‐3‐phosphoethanolamine (DPPE)‐Rhodamine]. 1,2‐distearoyl‐sn‐glycero‐3‐phosphoethanolamine‐N‐PEG2000‐dimenthylammonium propane/propanoyl (DSPE‐PEG[2000]‐DAP), the “adhesion lipid,” was custom synthesized by Avanti Polar lipids and characterized are previously reported.^18^ Vybrant® CFDA‐SE Cell Tracer Kit (CFDA‐SE), and 5/6‐fluorescein isothiocyanate (FITC) were purchased from ThermoFisher Scientific (Waltham, MA, USA). The PSMA‐targeting antibody was generously provided by Progenics Pharmaceuticals, Inc. Actinium‐225 (^225^Ac, actinium nitrate) was supplied by the U.S. Department of Energy Isotope Program, managed by the Office of Isotope R&D and Production. Indium‐111 (^111^In, indium chloride) was purchased from BWXT ITG Canada, Inc. (Vancover, B.C., Canada).

### 5.2 Liposome formation and loading

Liposomes were formed using the thin‐film hydration method as previously described.^32^ Briefly, lipids were combined at a mole ratio of 20PC:DPPS:Cholesterol:PEG(2000)‐DAP:DPPE‐Rhodamine 0.61:0.26:0.04:0.09:0.001 in a round bottom flask and dried on a rotovap. The film was then hydrated with citrate buffer (150 mM, pH 5.0), containing DOTA (12.5 mM) and ascorbic acid (23 mM) [for ^111^Indium‐loading, hydrated with phosphate buffered saline (PBS) containing 2 mM diethylenetriamine pentaacetic acid (DTPA)], and allowed to anneal at 75°C for 2 h, followed by extrusion 21 times through 100 nm pore polycarbonate membranes at 80°C. Finally, the liposomes were passed through a 10 cm Sepharose 4B column equilibrated with 4‐(2‐hydroxyethyl)‐1‐piperazineethanesulfonic acid buffer (pH 7.4, 20 mM HEPES, adjusted to 300 mOsm using sucrose). Liposome size distribution and zeta potentials were measured using a NanoSeries Zetasizer (Malvern Instruments Ltd, Worcestershire, UK).

Immediately after passing through the column, 1 ml of liposome suspension was heated to 75°C, and ^225^Ac‐Nitrate (dissolved in 0.2 N HCl) (or ^111^In‐Chloride), along with 25 μl of A23187 (15.6 mg/ml dissolved in ethanol) and 25 μl of metal‐free water, was added. After 1 h, the mixture was allowed to cool to room temperature, and after addition of ethylenediaminetetraacetic acid disodium salt dihydrate (EDTA), was passed through a Sephadex G‐50 column to remove unencapsulated radioactivity. Loading efficiency and content release were measured by comparing the activity counts before and after removal of released contents. For ^225^Ac, samples were allowed to reach secular equilibrium (6 h) before counting the γ‐photon emissions of Bismuth‐213 (360–480 keV γ‐emission) using a γ‐counter (Packard Cobra II Auto‐Gamma, Model E5003). For ^111^In, the 100–400 keV γ‐emissions were measured.

### 5.3 Antibody radiolabeling

After thorough washing, the antibody was resuspended in 0.1 M sodium carbonate buffer (pH 9.0) at a concentration of 2.5 mg/ml and placed on ice. DOTA‐SCN (dissolved in dimethylformamide at 10 mg/ml) was added dropwise until a 40:1 chelator:antibody mole ratio was reached. (For ^111^In‐labeling, DTPA‐SCN dissolved in dimethyl sulfoxide at 20 mg/ml was added dropwise at a 15:1 chelator:antibody ratio). The mixture was kept on ice and allowed to shake overnight. The mixture was then passed through a 10DG column (equilibrated with 0.1 M Tris–HCl buffer, pH 9.0 for ^225^Ac‐radiolabeling or 1 M acetate buffer, pH 4.5 for ^111^In‐radiolabeling) to remove any unconjugated chelator. The final concentration of antibody after column was confirmed using the Pierce bicinchoninic acid assay. For radiolabeling, to 0.1 mg of DOTA‐SCN‐labeled (or DTPA‐SCN‐labeled) antibody suspended in 0.5 ml of Tris–HCl buffer (or acetate buffer), 3 μl of radioactivity in 0.2 N HCl was added and was incubated at 37°C for 1 h. Finally, after addition of EDTA, the mixture was passed through a 10DG column (equilibrated with PBS, pH 7.4) to remove any unchelated radionuclides. Radiolabeling yield was calculated as the ratio of the measured radioactivity before and after the final 10DG column.

Radiopurity was measured using instant thin layer chromatography as previously reported using 10 mM EDTA as the mobile phase.^33^ Purity was calculated as the percent of radioactivity remaining at the bottom quarter of the strip relative to top half. Immunoreactivity was measured by incubating cells (1 million cells/ml) with radiolabeled antibody at a 100:1 receptor:antibody ratio on ice for 1 h until equilibrium was reached, after which it was washed three times with ice cold PBS. A second tube, containing a 50 times excess of unlabeled antibody was run in parallel to account for nonspecific binding. Immunoreactivity was calculated as the ratio of bound radiolabeled antibody to the initial amount of radioactivity added, corrected for the nonspecific binding.

### 5.4 Cell culture and spheroid formation

The LNCaP cell line was purchased from ATCC. The C4‐2B and PC3 cell lines were obtained from the Pienta lab (Dr. Sarah Amend). The PC3‐PIP cell line was obtained from the Pomper lab (Dr. Sangeeta Ray, obtained from Dr. Warren Heston [Cleveland Clinic]). LNCaP, PC3, and C4‐2B cell lines were cultured in RPMI media supplemented with 10% FBS and 1% Pen‐Strep. The PC3‐PIP cell line was cultured in the same, with 20 μg/ml Puromycin added to maintain PSMA expression.

For spheroid formation, Corning ultra‐low attachment surface 96‐well round‐bottom plates were used; for the LNCaP and C4‐2B cell lines, 50 cells/well were plated, and for the PC3 and PC3‐PIP cell line, 200 cells/well were plated and centrifuged at 2500 RPM for 5 min, before allowing spheroids to reach the desired size.

### 5.5 Evaluation of microdistributions of carriers in spheroids

Spheroids were incubated with CFDA‐SE‐encapsulating liposomes (2 mM lipid, 0.8 μM CFDA‐SE), containing DPPE‐Rhodamine (ex/em: 550/590 nm), or with FITC‐labeled PSMA‐targeting antibody (0.06 μM, corresponding to 100× excess of PSMA receptors expressed by cells per spheroid) for times corresponding to their treatment incubation times (6 h with liposomes, 24 h with the antibody, vide infra). CFDA‐SE (ex/em: 497/517 nm) was used as the hydrophilic drug surrogate, antibodies were fluorescently labeled with FITC (ex/em: 494/518 nm) as reported before.^34^ At various time points during incubation with the carriers (uptake) and after removal of the carriers from the surrounding media (clearance), different spheroids were sampled, frozen in OCT gel, sliced in 20 μm thick sections, and the equatorial slices were imaged using a confocal fluorescence microscope (Zeiss LSM 780 using a 10× objective). An in‐house Matlab erosion code was then used to quantify the radial spatial distributions of the fluorescent labels (of 5 μm concentric rings), and the trapezoid rule was used to calculate the time‐integrated concentrations.

### 5.6 Spheroid treatment and outgrowth

Spheroids (one per well) of 200, 400, and 600 μm in diameter were incubated with fixed total radioactivity concentrations while varying the radioactivity ratio between the radiolabeled antibody and the liposomes. The total mass of antibody added corresponded to 100 times excess of PSMA receptors expressed per spheroid. The radiolabeled antibody was added to each well for 18 h, after which the corresponding amount of liposomes was added for an additional 6 h (24 h total exposure to antibody). The incubation times for each carrier were chosen so as to approximately scale with their half‐lives of circulation in mice. Spheroids were then transferred into fresh media (one spheroid per well), and their size was monitored until the no‐treatment condition reached a plateau of about 800–900 μm in‐diameter.

At that point, each spheroid was transferred to a cell culture‐treated flat bottom well. When the cells of the no‐treatment condition reached confluency, the cells in each well were counted. The percent outgrowth was calculated as the number of cells in each treatment condition divided by the number of cells in the no‐treatment condition.

### 5.7 Colony formation assay

In six‐well plates, 500,000 cells/well were plated for 24 h and then the media was replaced with fresh media at the desired pH, and the radioactive constructs were added at varying radioactivity concentrations (ranging from 74 to 1 kBq/ml). Following 6 h of incubation, each well was washed thrice with PBS to ensure all radiation was removed. Cells were then scraped and suspended in fresh media, and were then plated at varying cell densities (ranging from 100 cells/5 ml [20 cells/ml] to 50,000 cells/20 ml [2500 cells/ml]) in Petri dishes. These dishes were placed in an incubator and allowed to grow for approximately 10 doubling times until measurable colonies were formed from surviving cells. Following this, each dish was stained with crystal violet, and the number of colonies formed was counted. Accounting for the initial cell plating density, the survival fraction was calculated at each radioactivity concentration by normalizing by the no‐treatment condition.

### 5.8 Tumor growth inhibition study

Male, NSG (NOD scid gamma) mice (purchased from JHU), 6–8 week old and 20–24 g weight, were inoculated in the hind flank with 200,000 PC3‐PIP cells suspended in 100 μl 1:1 v:v RPMI:Matrigel. The tumors were allowed to grow (approximately 2 weeks) until they reached a volume of 25–50 mm^3^, at which point mice were randomly assigned to a treatment condition.

Mice were intravenously injected 100 μl of either 4.63 kBq of ^225^Ac‐DOTA encapsulated in tumor‐responsive liposomes, 4.63 kBq of ^225^Ac‐DOTA‐SCN‐labeled PSMA‐targeting antibody (15 μg total mass of antibody per 20 g mouse), or a combination of both at same total administered radioactivity (with the ratio of hot:cold antibody kept constant between different ratios, i.e., 7.5 μg total mass of antibody for the 50:50 condition). Following treatment, the tumor volume was measured with a digital caliper (resolution 0.01 mm) and mouse weight was tracked daily. After 14 days post‐treatment, when not treated mice began to lose 10% of their initial body weight, all mice were euthanized and dissected, and histopathology analysis was performed on H&E stained sections of all organs and tumors. All mouse experiments were performed in accordance with protocols approved by the Johns Hopkins University Institutional Animal Care and Use Committee.

### 5.9 Biodistributions of the carriers

Tumor‐bearing mice were intravenously injected with 100 μl of 370 Bq of ^111^In‐DTPA encapsulated in tumor‐responsive liposomes or of ^111^In‐DTPA‐SCN‐labeled PSMA‐targeting antibody (diluted to a total mass of 7.5 μg antibody, corresponding to the 50:50 condition). At different time points, mice were euthanized and each organ was weighed and measured on a gamma counter for radioactivity. The measured activities were decay corrected to the time of injection, and the percent of initial injected activity per organ weight (%IA/g) was calculated.

### 5.10 Dosimetry

Dosimetry was performed using the methodology described in ref 35 using the biodistributions of ^111^In‐labeled tumor‐responsive liposomes and of the ^111^In‐labeled PSMA‐targeting antibody. ^111^In has been confirmed as a surrogate of ^225^Ac biodistributions delivered by internalizing antibodies and by our liposomes.^16^, ^36^ Briefly, following decay correction and multiplication by ^225^Ac half‐life decay factor, the data were integrated to generate the time‐integrated activity coefficients (TIACs) (using the software package 3D‐RD‐S, Radiopharmaceutical Imaging and Dosimetry, LLC [Rapid], Baltimore MD). The resulting TIACs were then multiplied by the energy associated with α‐particle and electron emissions of ^225^Ac and its daughters (ICRU decay scheme). For liposomes, 25% of the ^213^Bi generated in the tumor from ^225^Ac decays was assumed to decay in the kidneys as previously reported.^16^ For the PSMA‐targeting antibody, all ^213^Bi generated in the tumor were assumed to be retained in the tumor.

### 5.11 Tumor α‐camera imaging

Tumor‐bearing mice were injected with 148 kBq of either tumor‐responsive liposomes encapsulating ^225^AcDOTA, ^225^AcDOTA‐SCN‐labeled antibody (total mass of antibody 15 μg per animal), or a 80:20 ratio of liposomes: antibody (total mass of antibody 12 μg per animal). Mice were euthanized 24 h after injection, and tumors (75 mm^3^) were removed and allowed to reach secular equilibrium for 6 h. Tumors were sliced into 16 μm sections, which were placed on scintillation paper for α‐camera imaging as previously reported.^16^

### 5.12 Toxicity study

Tumor‐free 6–8‐week‐old male NSG (NOD scid gamma) mice were injected with 4.63 kBq and 2.31 kBq of ^225^Ac‐DOTA‐SCN‐labeled antibody. One month post‐injection, all mice were euthanized and organs were H&E stained for histopathology evaluation. The MTD of ^225^Ac delivered by tumor‐responsive liposomes on the same animal strain was previously found to be 5.55 kBq per 20 g animal.^16^

### 5.13 Statistical analysis

All results are reported as the mean ± standard deviation between *n* independent measurements (specifics for each experiment stated in each figure captions). Significance between treatment conditions was evaluated using one‐way analysis of variance and the unpaired Student's t‐test, with significance defined as *p* < 0.05.

## AUTHOR CONTRIBUTIONS

**Dominick Salerno:** Data curation (lead); formal analysis (supporting); visualization (supporting); writing – original draft (supporting). **Alaina Howe:** Data curation (supporting). **Omkar Bhatavdekar:** Data curation (supporting). **Anders Josefsson:** Data curation (supporting). **Jesus Pacheco‐Torres:** Data curation (supporting); formal analysis (supporting). **Zaver Bhujwalla:** Data curation (supporting); formal analysis (supporting). **Kathleen L. Gabrielson:** Formal analysis (supporting). **Stavroula Sofou:** Conceptualization (lead); formal analysis (lead); funding acquisition (lead); investigation (lead); methodology (lead); project administration (lead); resources (lead); supervision (lead); validation (lead); visualization (equal); writing – original draft (lead); writing – review and editing (lead).

## CONFLICT OF INTEREST

The authors have no conflicts of interest to declare.

## Supporting information


**Appendix** **S1**: Supporting Information

## Data Availability

The data that support the findings of this study are available from the corresponding author upon reasonable request.
