# Supplementary material for: Two diverse carriers are better than one: A case study in α‐particle therapy for prostate specific membrane antigen‐expressing prostate cancers
Source: Bioeng Transl Med. 2021 Nov 17;7(2):e10266. doi: 10.1002/btm2.10266 (PMC9115683; doi:10.1002/btm2.10266)
Supplement: Supplementary file 1 — Appendix S1: Supporting Information [file BTM2-7-e10266-s001.pdf]

SUPPLEMENTAL INFORMATION

Materials

Cholesterol, Trisodium citrate dihydrate, anhydrous citric acid, calcium ionophore A23187, 1,4,7,10-Tetraazacyclododecane-1,4,7,10-tetraacetic acid (DOTA), ascorbic acid, 4-(2-hydroxyethyl)-1-piperazineethanesulfonic acid (HEPES), sucrose, sodium carbonate, sodium acetate, acetic acid, Dimethyl sulfoxide (DMSO), Dimethylformamide (DMF), Phosphate Buffered Saline (PBS), Sephadex G-50, Sepharose-4B were purchased from Sigma-Aldrich (Atlanta, GA, USA). Ethylenediamine tetraacetic acid, Disodium salt dihydrate (EDTA) and SNARF-4F were purchased from Fisher Scientific (Pittsburgh, PA, USA). S-2-(4-Isothiocyanatobenzyl)-1,4,7,10-tetraazacyclododecane tetraacetic acid (DOTA-SCN) and S-2-(4-Isothiocyanatobenzyl)-diethylenetriamine pentaacetic acid (DTPA-SCN) were purchased from Macrocyclics (Dallas, TX, USA). Penicillin-Streptomycin and Puromycin Dihydrochloride were purchased from ThermoFisher Scientific (Waltham, MA, USA). Trypsin and Matrigel™ were purchased from Corning (Corning, NY, USA). Roswell Park Memorial Institute (RPMI) 1640 Medium was purchased from ATCC (Manassas, VA, USA). Fetal Bovine Serum (FBS) was purchased from Omega Scientific (Tarzana, CA, USA). Chelex® resin, and chromatography columns were purchased from Bio-Rad (Hercules, CA, USA).

Dosimetry (verbatim from Methods in reference [1])

Dosimetry was performed based upon the methodology described in ref [2]. *Ex vivo*, activity concentration measurements for normal organs and the tumor were collected at different time points post administration of ^{111}In-labeled tumor-responsive liposomes or the ^{111}In-labeled PSMA-targeting antibody (n=3 for at each time-point). ^{111}In has been confirmed as a surrogate of

^{225}Ac biodistribution [3]. These data were decay corrected to the time of injection and multiplied by a decay factor corresponding to the ^{225}Ac half-life at each time point to give time vs. activity curves for ^{225}Ac-labeled- liposomes and antibody. The data were then integrated over time using a combination of numerical (trapezoidal) and analytical integration of fitted exponential expressions (as implemented in the software package 3D-RD-S, Radiopharmaceutical Imaging and Dosimetry, LLC (Rapid), Baltimore MD). The resulting time integrated activity coefficients (TIACs) were multiplied by the energy associated with alpha particle and electron emissions of ^{225}Ac and its daughters (ICRU decay scheme) to obtain the absorbed dose for each tissue (see equations 1 and 2). This calculation assumes that all alpha-particle and electron energy is absorbed by each source tissue. For liposomes, seventy-five percent of the ^{213}Bi generated in the tumor from ^{225}Ac decays was assumed retained in the tumor; the remaining 25% was assumed to decay in the kidneys. These percentages were arrived at based upon the diffusion properties of a charged atom in tissue where it is likely to associate with the nearest protein.

In particular, liposomes were designed to not be significantly internalized by tumor cells; therefore, from all emissions of ^{225}Ac and its daughters, that occurred in the interstitial space in the vicinity of cells , 50% of generated ^{213}Bi was estimated to remain within cells, as previously demonstrated [4] and, for the other 50%, about half was estimated to escape the tumor using the following rationale: The root mean squared displacement of the longest lived daughter, ^{213}Bi , when associated with a small protein (30kDa with $D=100\mu m^2/sec$ [5]) is 0.9 mm in 90 minutes (equal to two half-lives of ^{213}Bi). This distance is overestimated since it is true for diffusion in a free medium and not in pores (as is the case in the tumor interstitium). Nevertheless, the characteristic length of tumors in our studies at the time of the administration of therapy was of

the order of 1.8 mm. Therefore, half of ^{213}Bi that was generated in the pores (0.5 x 50% of ^{213}Bi) was assumed to escape the tumor.

$$D_{\alpha+e^-}(r_T) = \tilde{A}(r_T) \cdot \frac{\sum_i \Delta_i^{\alpha+e^-} \phi(r_T \leftarrow r_S; E_i^{\alpha+e^-})}{M(r_T)} \qquad \text{(Eq. 1)}$$

$$\phi(r_T \leftarrow r_S; E_i^{\alpha+e^-}) = \begin{cases} 1 & r_T = r_S \\ 0 & r_T \neq r_S \end{cases} \qquad \text{(Eq. 2)}$$

$\sum_i \Delta_i^{\alpha+e^-} = 8.13 \times 10^{12}$ (Gy·kg)/(Bq·s) for ^{225}Ac and all daughters

$\sum_i \Delta_i^{\alpha+e^-} = 5.05 \times 10^{12}$ (Gy·kg)/(Bq·s) for ^{213}Bi and all daughters

$\tilde{A}(r_T)$ – time integrated activity in target region, *T*

$D_{\alpha+e^-}(r_T)$ – absorbed dose to target region, *T*, from alpha-particle and electron emissions

$\phi(r_T \leftarrow r_S; E_i^{\alpha+e^-})$ – fraction of alpha-particle or electron energy from emission *i* in source region *S* that is absorbed in target region, *T*.

$\Delta_i^{\alpha+e^-}$ - alpha-particle or electron energy per radionuclide disintegration from emission *i*

$M(r_T)$ – mass of target region, *T*.

PCa Cell Line	PSMA receptor copies per cell	Measured K_D (PSMA-Targeting Antibody ^{111}In-DTPA-SCN-Ab)	Cells' Doubling Time (hours)
PC3	no measureable expression	N/A	18
C4-2B	126,000 ± 12,000	14.1 ± 3.5 nM	24
LNCaP	210,000 ± 12,000	7.2 ± 1.5nM	36
PC3-PIP	3,400,000 ± 78,000	28 ± 1.6 nM	16

TABLE S1 Characterization of prostate cancer cell lines: PSMA expression (results from Figure S2), doubling time, and the measured K_D of the PSMA-targeting antibody.

(a) PSMA-targeting ^{111}In-DTPA-SCN-antibody

	%IA/g					
Time (hours):	1	8	24	48	72	96
Blood	15.82 ± 6.88	11.47 ± 1.70	8.92 ± 2.11	4.83 ± 0.99	2.98 ± 0.14	2.06 ± 0.51
Heart	4.53 ± 1.75	3.66 ± 0.63	1.74 ± 0.35	0.91 ± 0.05	0.86 ± 0.17	0.45 ± 0.07
Lungs	2.58 ± 1.42	5.26 ± 4.23	1.40 ± 0.96	0.58 ± 0.26	0.89 ± 0.55	0.62 ± 0.16
Liver	4.16 ± 3.41	7.17 ± 3.66	9.30 ± 4.49	3.97 ± 0.46	4.37 ± 3.75	3.41 ± 4.35
Spleen	5.21 ± 3.03	7.33 ± 2.30	13.12 ± 2.53	14.92 ± 4.61	17.35 ± 6.50	7.27 ± 3.19
Stomach	0.53 ± 0.52	0.84 ± 0.23	0.52 ± 0.06	0.50 ± 0.16	0.13 ± 0.06	0.14 ± 0.05
Intestines	1.53 ± 0.34	5.87 ± 1.25	3.40 ± 1.06	0.91 ± 0.70	1.06 ± 0.39	1.01 ± 0.65
Kidneys	5.74 ± 2.31	8.78 ± 1.41	3.27 ± 0.77	1.59 ± 1.31	1.83 ± 0.22	1.14 ± 0.57
Prostate	0.20 ± 0.27	0.18 ± 0.02	0.39 ± 0.18	0.38 ± 0.12	0.05 ± 0.06	0.16 ± 0.13
Muscle	0.03 ± 0.01	0.05 ± 0.01	1.66 ± 0.03	1.70 ± 0.47	1.09 ± 0.34	1.06 ± 0.46
Bone	0.86 ± 0.99	0.74 ± 1.22	3.57 ± 2.57	1.68 ± 1.65	1.12 ± 0.35	1.03 ± 0.94
PC3-PIP Tumor	0.43 ± 0.14	3.03 ± 0.92	3.32 ± 0.27	4.34 ± 1.08	1.50 ± 2.05	1.00 ± 0.85

(b) ^{111}In-DTPA encapsulating liposomes

	%IA/g				
Time (hours):	1	16	24	32	48
Blood	10.26 ± 2.44	4.45 ± 0.49	2.87 ± 0.72	1.42 ± 0.17	0.93 ± 0.20
Heart	2.51 ± 1.18	0.49 ± 0.17	0.25 ± 0.03	0.12 ± 0.01	0.11 ± 0.02
Lungs	2.75 ± 0.42	0.62 ± 0.12	0.96 ± 0.44	0.44 ± 0.15	0.06 ± 0.01
Liver	7.51 ± 0.75	8.04± 1.61	10.86 ± 3.73	6.10 ± 0.96	3.93 ± 2.28
Spleen	6.16 ± 1.41	7.87 ± 4.98	12.42 ± 5.40	14.08 ± 5.68	6.89 ± 3.89
Stomach	0.87 ± 0.32	0.43 ± 0.02	0.54 ± 0.30	0.28 ± 0.20	0.16 ± 0.03
Intestines	1.79 ± 0.33	0.90 ± 0.22	0.93 ± 0.08	0.36 ± 0.17	0.31 ± 0.26
Kidneys	2.58 ± 1.20	1.91 ± 1.87	1.57 ± 0.97	1.68 ± 0.62	1.47 ± 0.38
Prostate	0.37 ± 0.43	0.85 ± 0.91	0.17 ± 0.13	0.31 ± 0.14	0.03 ± 0.02
Muscle	0.67 ± 0.64	0.20 ± 0.21	0.32 ± 0.15	0.28 ± 0.14	0.39 ± 0.10
Bone	0.33 ± 0.14	0.44 ± 0.14	1.40 ± 0.69	0.26 ± 0.22	0.48 ± 0.21
PC3-PIP Tumor	0.58 ± 0.39	1.10 ± 0.93	3.59 ± 0.89	1.85 ± 0.25	1.11 ± 0.36

TABLE S2 Table of average and standard deviations (between n=3 mice per time point per condition) of biodistributions of (a) the PSMA-targeting ^{111}In-DTPA-SCN-antibody and (b) ^{111}In-DTPA encapsulating tumor-responsive liposomes, in PC3-PIP tumor bearing NSG mice.

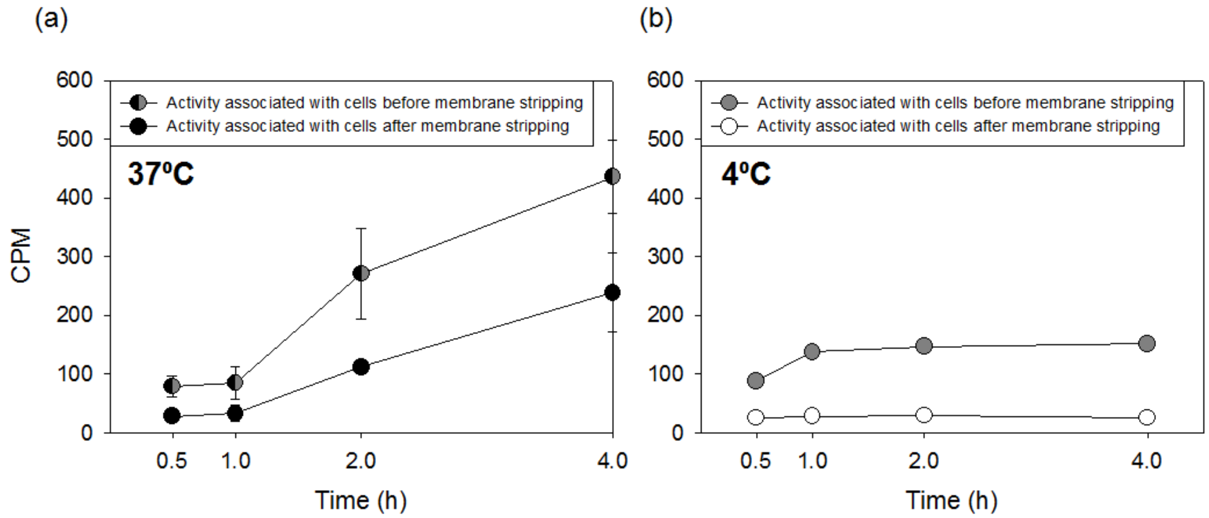

FIGURE S1 Binding and/or internalization of the PSMA-targeting ^{225}Ac-DOTA-SCN-antibody (740kBq/mg) incubated with PC3-PIP prostate cancer cells (a) at 37ºC and (b) on ice. Error bars correspond to the standard deviation of two samples per condition. Lines only serve as guide to the eye.

Method

Cells (2 million cells/mL, n=2 samples per condition) were incubated (in a humidified incubator at 37ºC or on ice) for 0.5, 1, 2, and 4 hours with the radiolabeled antibody. At each time point, 1mL of the parent cell suspension was removed, washed and measured for radioactivity before and after stripping away the surface bound antibody by washing cells with acidic glycine buffer (50 mM glycine/150 mM NaCl, pH 3.0) for 5 minutes at room temperature. In all cases, radioactivity was measured on a Gamma counter (360-480keV) after reaching secular equilibrium (6 hours) and correcting the counts measured for the number of cells on the sampled suspension.

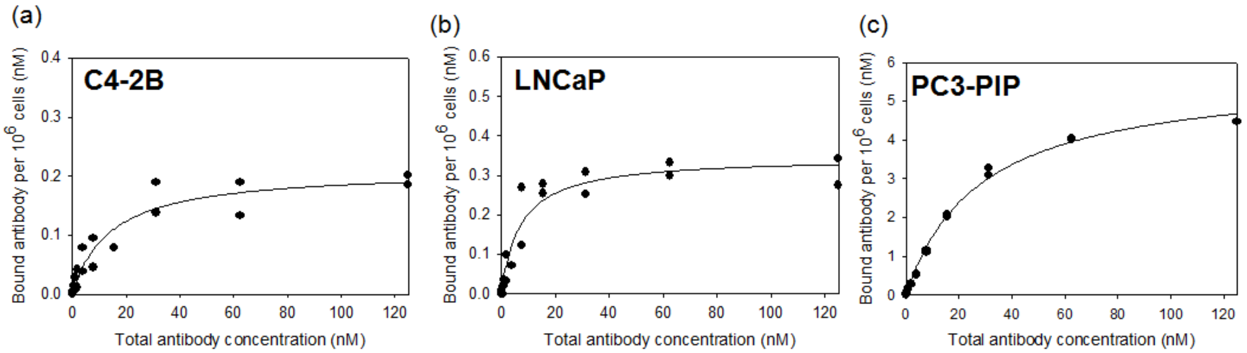

FIGURE S2 Binding curves of the PSMA-targeting ^{111}In-DTPA-SCN-antibody with the following prostate cancer cell lines: (a) C4-2B, (b) LNCaP, and (c) PC3-PIP. Immunoreactivity of the radiolabeled antibody was 71%. Incubation of cells with the antibody was performed on cell suspensions at a fixed cell density while constantly shaking at ice-cold temperature (2 samples per antibody concentration).

Method

Cells were suspended in tubes at 1 million cells/mL (in duplicate) and placed on ice. Varying concentrations of the PSMA-targeting ^{111}In-DTPA-SCN-antibody were added to each tube, and allowed to rotate on ice for one hour until equilibrium was reached. In parallel, a second set of tubes were pre-incubated for one hour with 30 times excess cold antibody to block the PSMA receptors, then was treated identically with the radiolabeled antibody. Upon completion of incubation, each tube was then washed three times using a refrigerated centrifuge and ice cold PBS, and the radioactivity bound to the cells was measured using a Gamma-counter. The nonspecific binding curve was subtracted, and a single rectangular hyperbola was fit to the resulting specific binding curve. The receptor expression and K_D were calculated from the fit parameters.

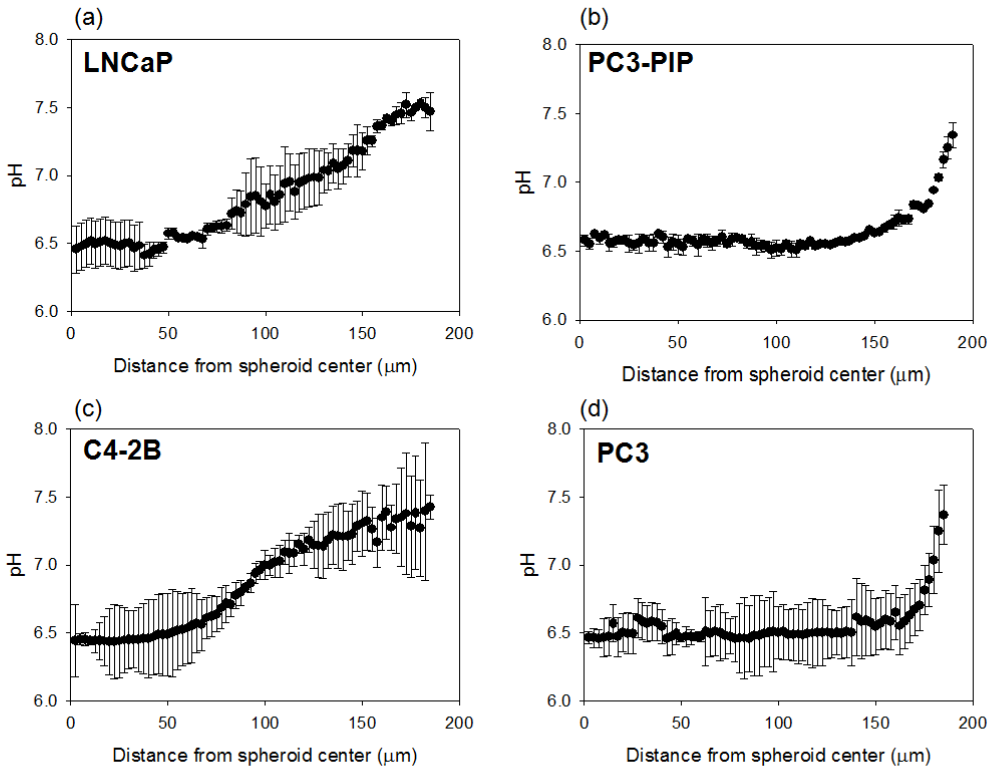

FIGURE S3 Spheroid interstitial pH profiles of (a) LNCaP, (b) PC3-PIP, (c) C4-2B, and (d) PC3 cell lines. Error bars correspond to the standard deviation of measurements between n=3-4 spheroids per cell line.

Method

The radial dependence of the interstitial pH of multicellular 400 μm-in-diameter spheroids was measured by incubating spheroids with the membrane impermeant pH indicator SNARF-4F in media overnight at 37°C. LNCaP and C4-2B spheroids were incubated at 500 μM whereas PC3 and PIP were incubated at 350 μM SNARF-4F. The ratio of SNARF-4F's emission intensities at 640 nm and 580 nm (ex: 514 nm) varies linearly with pH between the values 6 to 8 independent of the fluorophore's concentration. Right before measurement, spheroids were transferred into fresh media and optical slices (z=10 μm) were acquired immediately on Zeiss LSM 780. For

simplicity, only radial dependence was assumed. An in-house developed Matlab erosion algorithm (with concentric ring width equal to 5 μm) was applied on the images to evaluate the mean radial intensities and to generate the ratios of the intensities as a function of position. Calibration curve of SNARF-4F in media at known pH values was used to convert the ratios to pH values in spheroids. N=3-5 spheroids were imaged and averaged for the experiment.

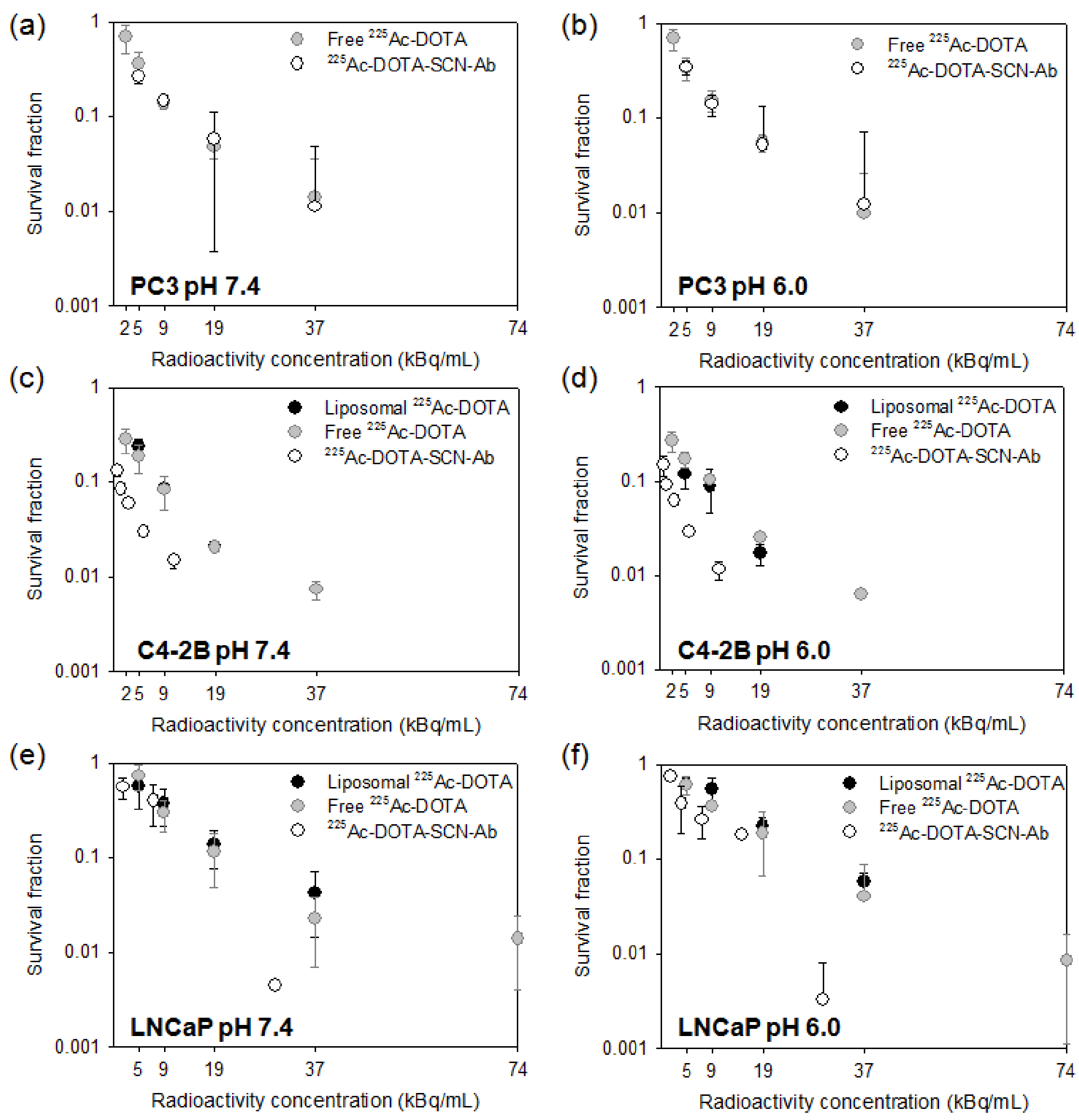

FIGURE S4 Colony survival of non-PSMA expressing PC3 (a, b), low PSMA-expressing C4-2B (c, d), and moderate PSMA-expressing LNCaP (e, f) cell lines following a 6 hour incubation in media at pH 7.4 (a, c, e) and pH 6.0 (b, d, f, representative of lowest expected extracellular pH value in the tumors' interstitium). Treatment with ^{225}Ac-loaded tumor-responsive liposomes (black symbols), free ^{225}Ac-DOTA (grey symbols), and PSMA-targeting ^{225}Ac-DOTA-SCN-antibody (white symbols). Error bars correspond to the standard deviation of repeated measurements (n = 3-6 samples per condition).

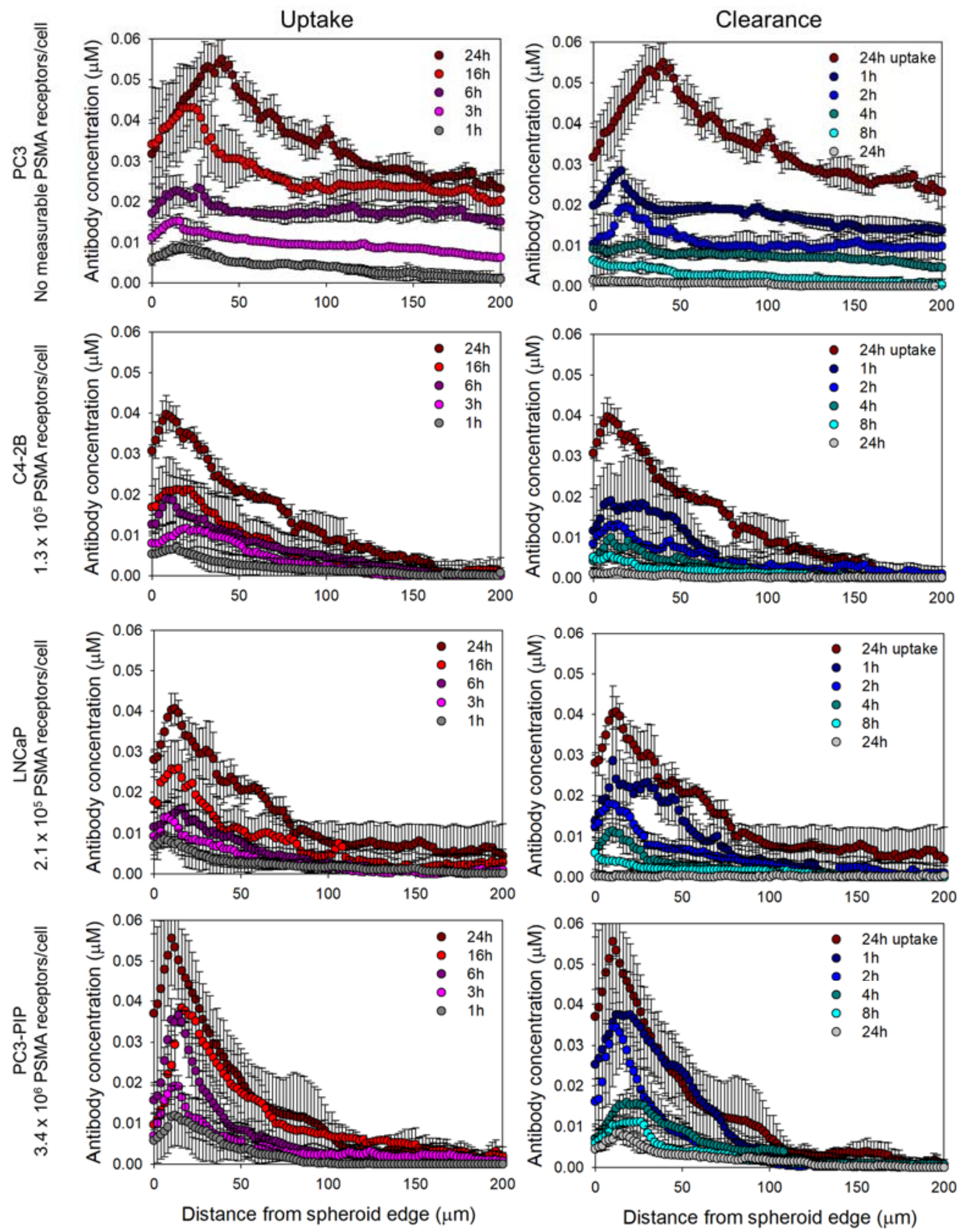

FIGURE S5 Uptake (left panel) and clearance spatial profiles (right panel) of the FITC-labelled PSMA-targeting antibody in 400 μm-in-diameter prostate cancer spheroids expressing different levels of the targeted receptor PSMA. Spheroids were incubated with the antibody for up to 24 hours. Errors correspond to standard deviations of repeated measurements (n = 3

spheroids). Spheroid diameters varied up to approximately 10%. Immunoreactivity of the fluorescently-labeled antibody was: 73 ± 5%.

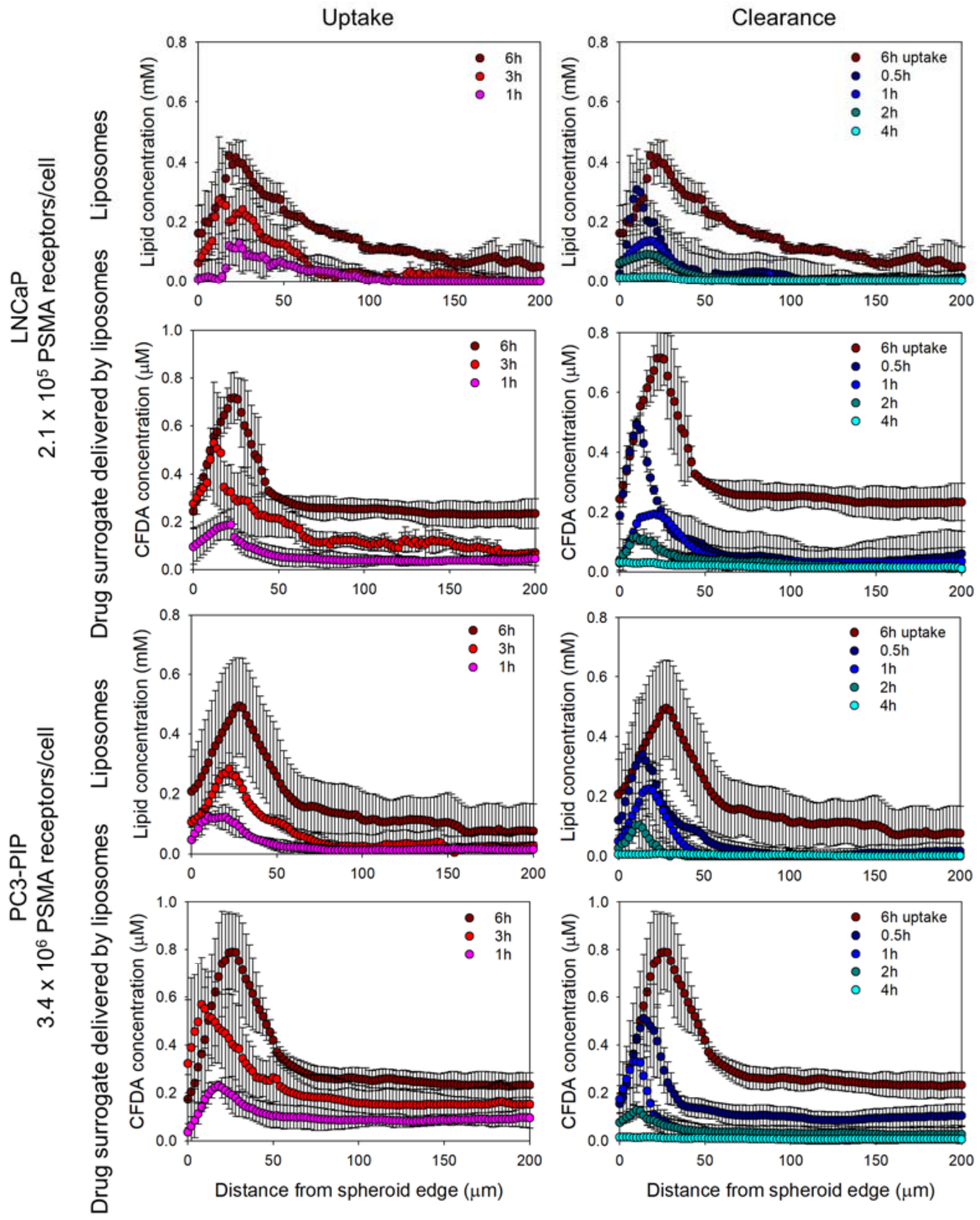

FIGURE S6 Uptake (left panel) and clearance spatial profiles (right panel) of the tumor-responsive DPPE-Rhodamine-liposomes and the drug surrogate, CFDA-SE, delivered by liposomes in 400 μm-in-diameter prostate cancer spheroids expressing different levels of the

targeted receptor PSMA. Spheroids were incubated with the liposomes for up to 6 hours. Errors correspond to standard deviations of repeated measurements (n = 3 spheroids). Spheroid diameters varied up to approximately 10%.

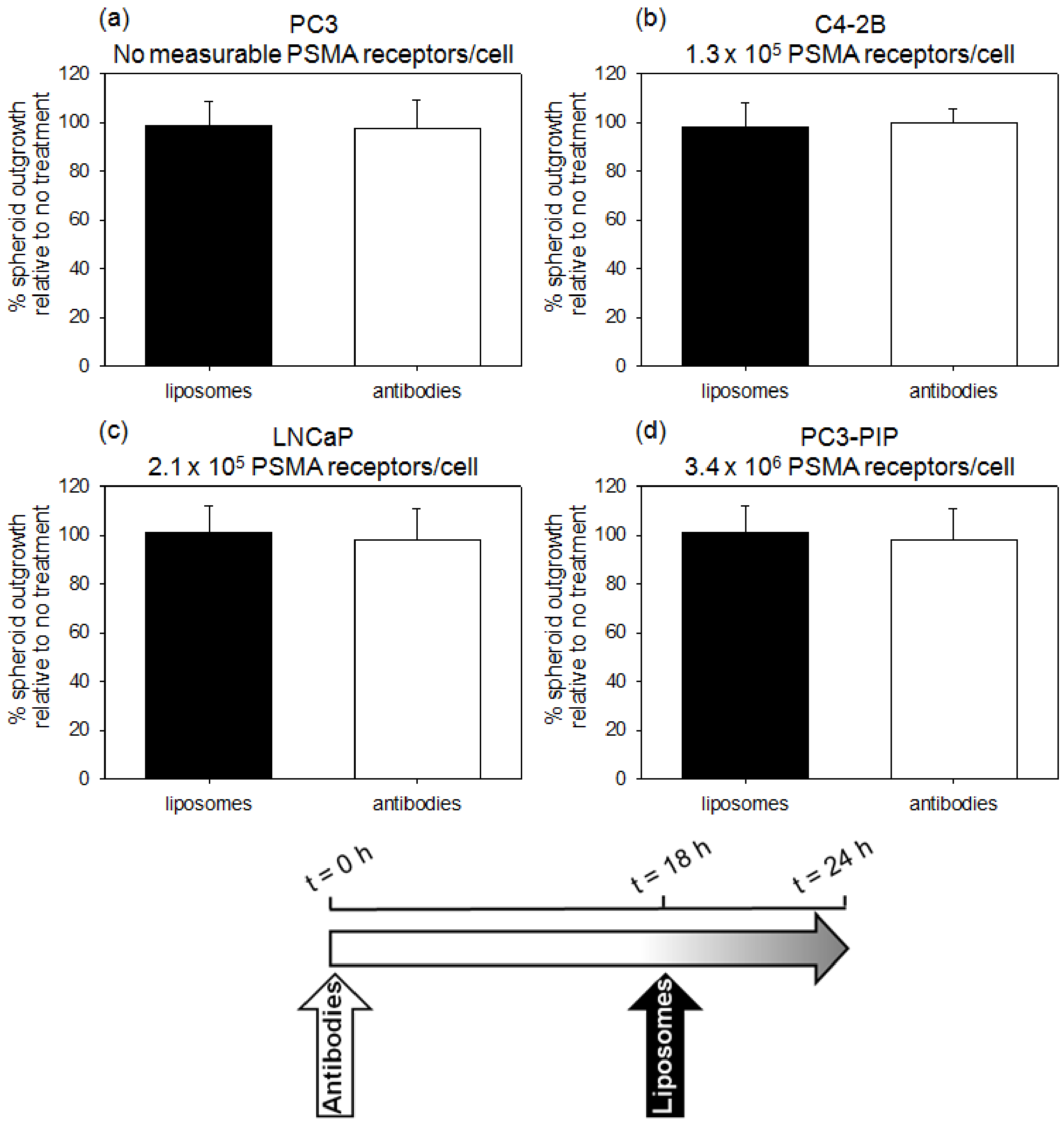

FIGURE S7 Treatment of spheroids with cold liposomes and the cold PSMA-targeting antibody. Error bars correspond to the standard deviation of repeated measurements (n = 6 spheroids per condition).

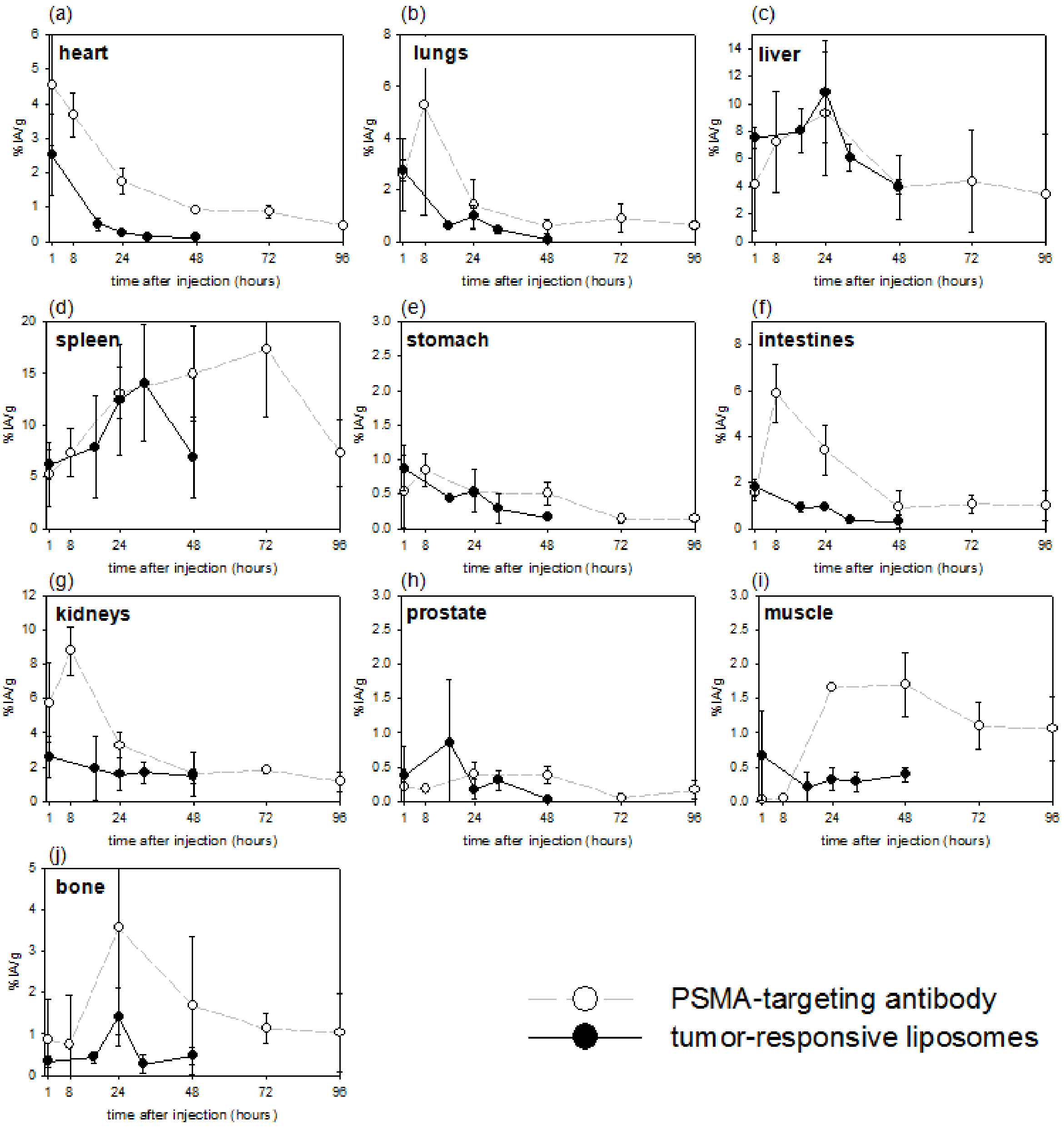

FIGURE S8 Biodistributions of PSMA-targeting ^{111}In-DTPA-SCN-antibody (white symbols) and ^{111}In-DTPA encapsulating tumor-responsive liposomes (black symbols) in PC3-PIP tumor bearing NSG mice: (a) heart, (b) lungs, (c) liver, (d) spleen, (e) stomach, (f) intestines, (g) kidneys, (h) prostate, (i) muscle, and (j) bone. Error bars correspond to the standard deviation of measurements averaged over n=3 mice per time point per condition. Numerical values are listed on Table S2.

(a)

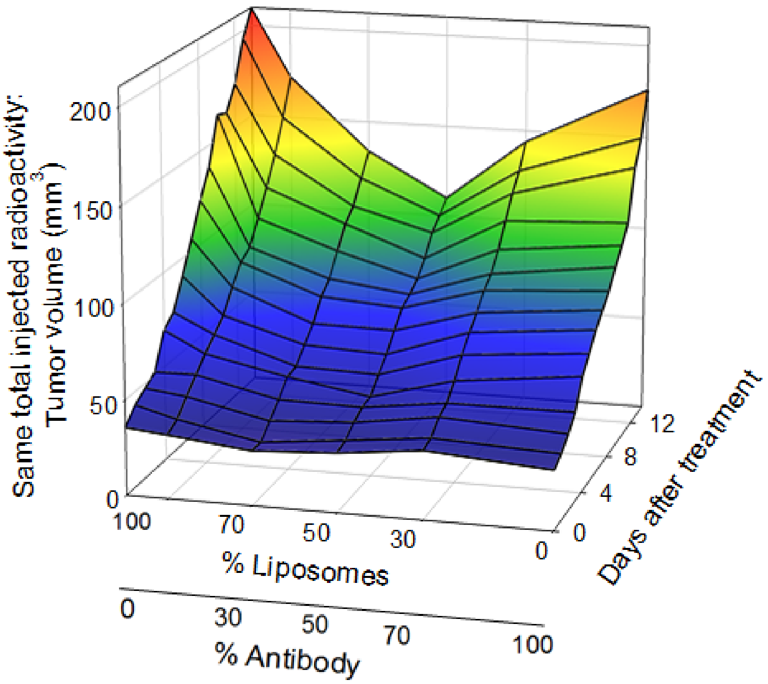

(b)

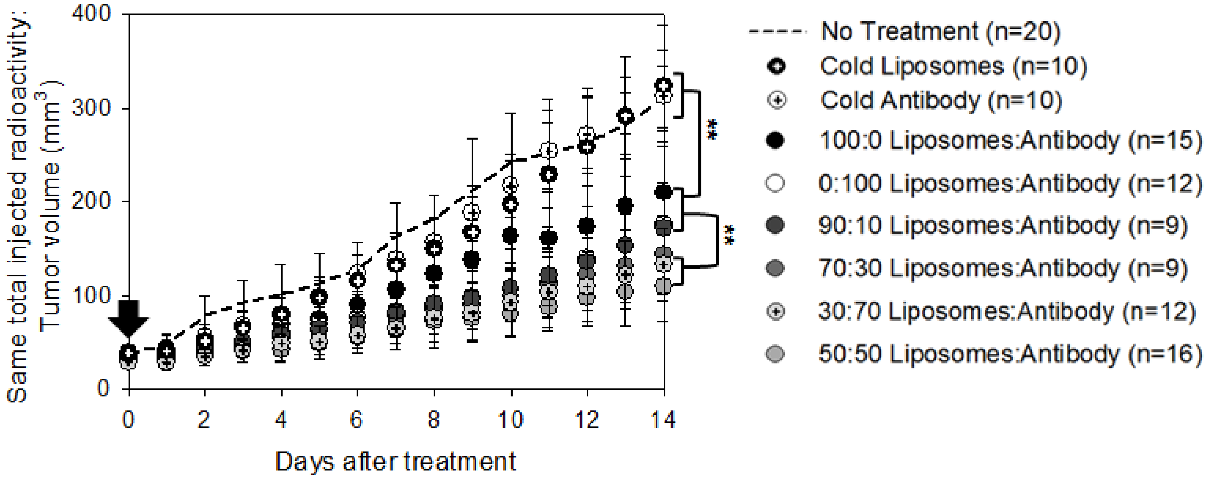

FIGURE S9 (a) 3D representation of PC3-PIP tumor volume as a function of time and the radioactivity split ratios between the two carriers. The non-treated and cold conditions are not shown on this plot.

(b) Tumor growth control response of PC3-PIP tumors following treatment with ^{225}Ac-DOTA encapsulating tumor-responsive liposomes (black symbols), PSMA-targeting ^{225}Ac-DOTA-SCN-

antibody (white symbols), and/or varying ratios of the two (grayscale symbols) at same total injected radioactivity. No treatment (dashed line) or tumor-responsive liposomes and PSMA-targeting antibody without ^{225}Ac (black/white symbols with cross sign) are also shown, with no difference between the three. Error bars correspond to the standard deviation of measurements averaged over n mice per condition (n = number of mice per treatment condition indicated in legend). * indicates *p-values* < 0.05, ** *p-values* < 0.01.

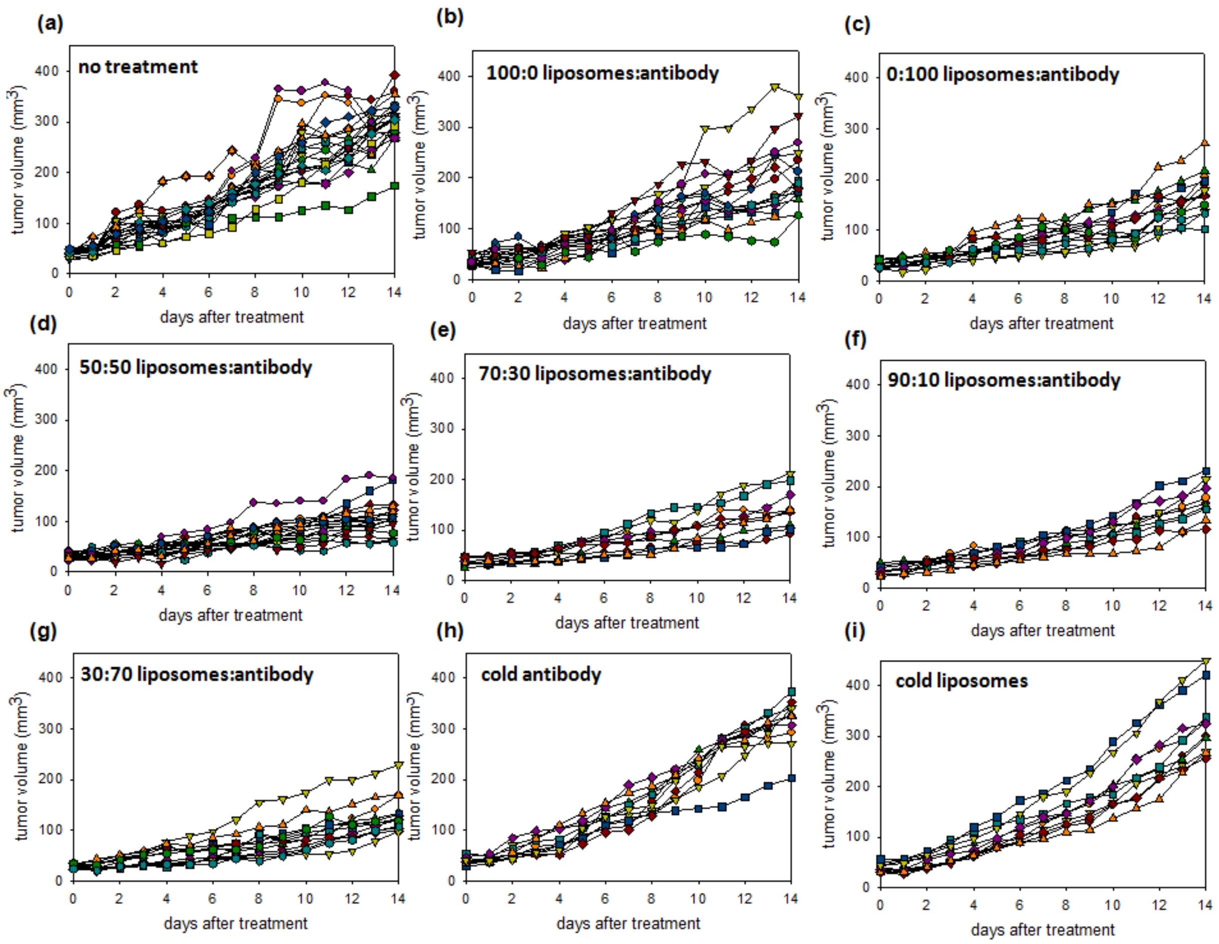

FIGURE S10 Individual tumor growth plots for each mouse in each treatment condition: (a) no treatment, (b) 100:0, (c) 0:100, (d) 50:50, (e) 70:30, (f) 90:10, (g) 30:70 liposomes:antibody, (h) cold antibody and (i) cold liposomes.

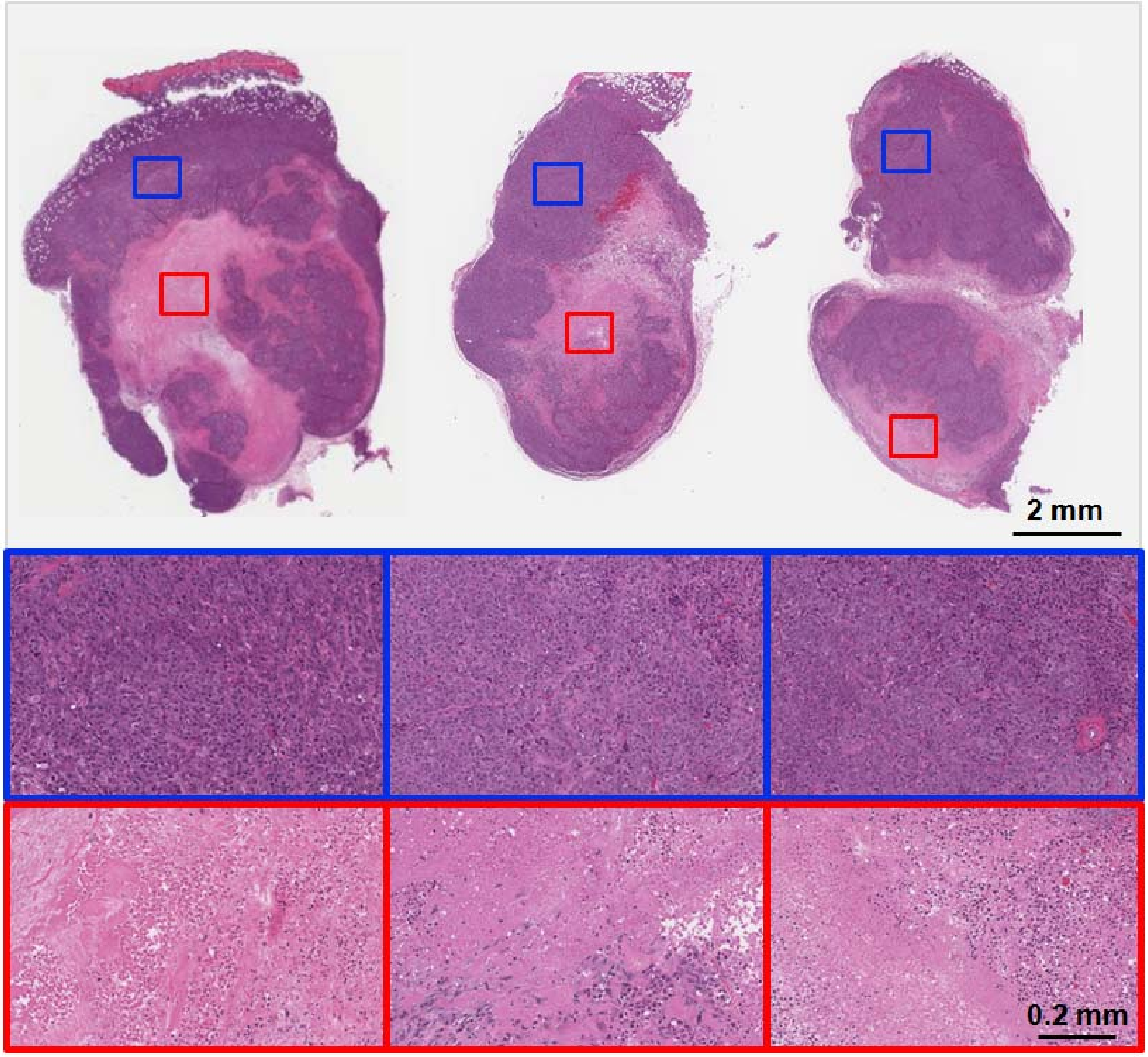

FIGURE S11 H&E images of non-treated PC3-PIP tumors. The images show large regions of necrotic tissue. This was attributed to the aggressive nature of this tumor model.

The tumor necrotic regions are indicated by the red frames. The blue frames indicate tumor regions that contain cancer cells and probably vasculature.

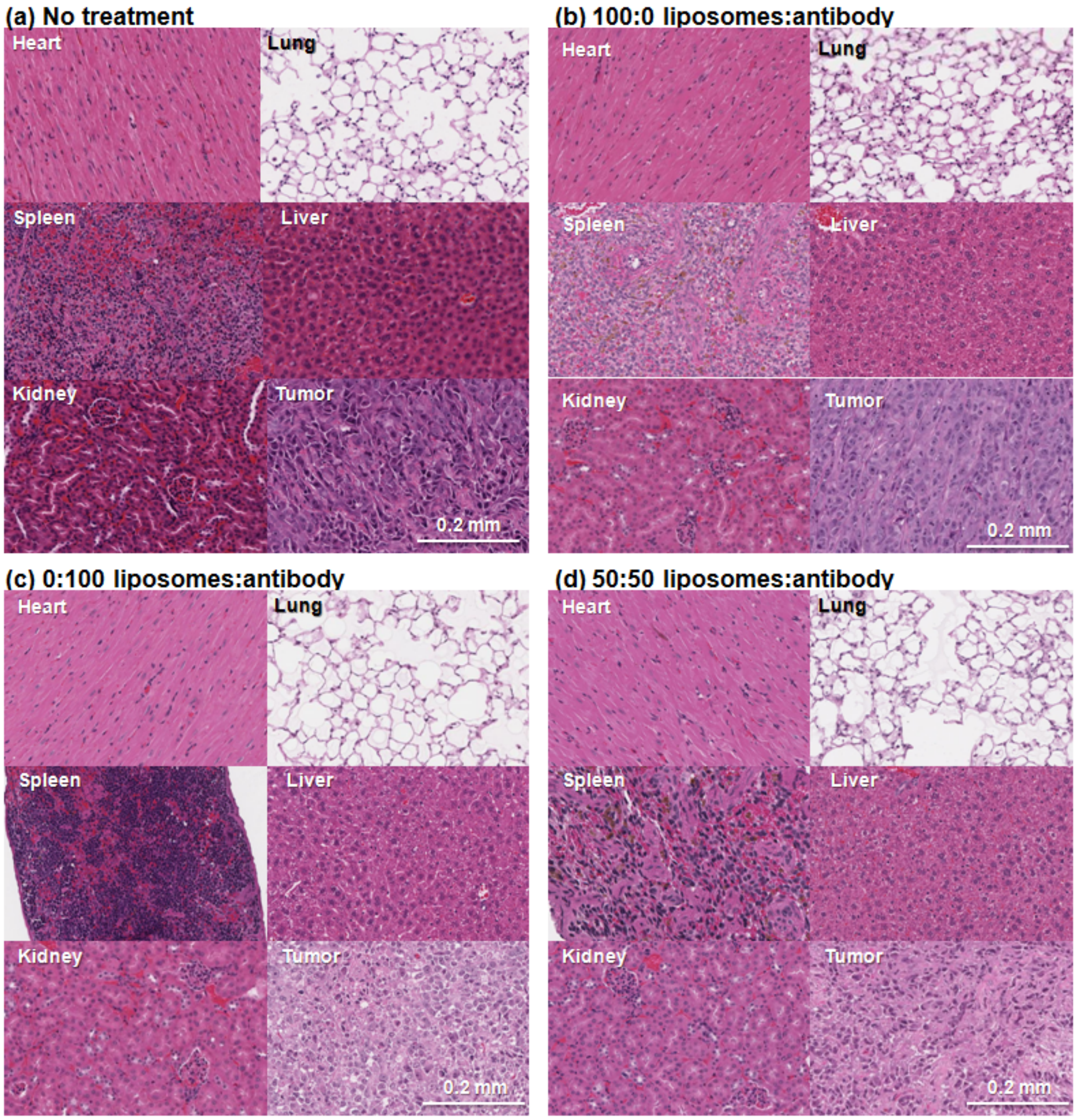

FIGURE S12 Treatment study: Normal tissue and tumor H&E images from PC3-PIP tumor-bearing NSG male mice treated with tumor-responsive liposomes loaded with ^{225}Ac-DOTA and/or the PSMA-targeting ^{225}Ac-DOTA-SCN-antibody at a total (single I.V.) dose equal to 4.63 kBq per 20g animal. The spleen of mice treated with α-particles showed low levels of extramedullary hematopoiesis (EMH), which is to be expected as the spleen was observed as a major site of both liposome and antibody uptake. As previously shown in MTD studies with these tumor-responsive liposomes, the reduced EMH is expected to reverse post treatment, and spleens were fully recovered after 9.5 months when tumor-free mice were treated at this dose [1].

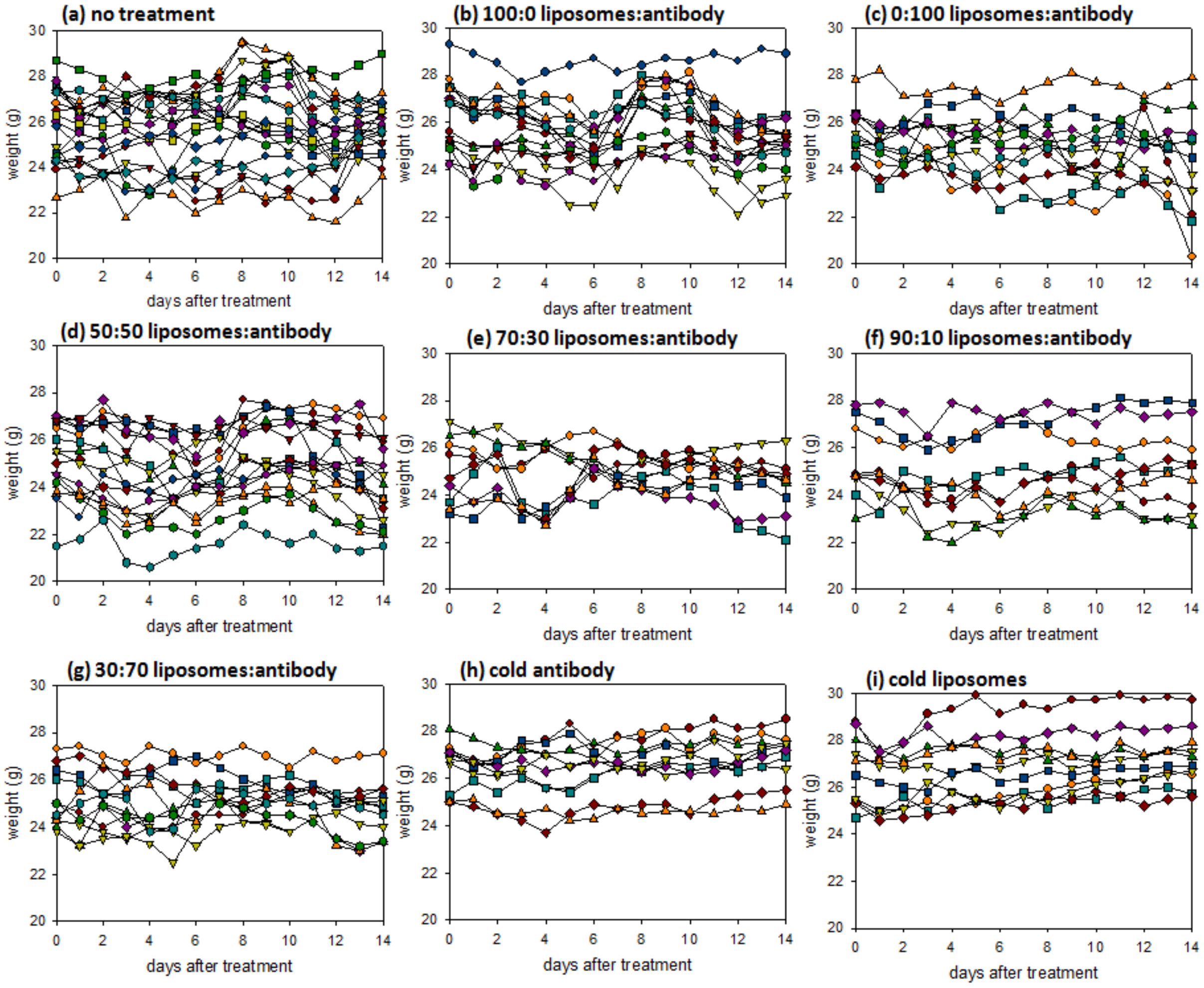

FIGURE S13 Treatment study: Individual mouse weight tracking plots for animals in each treatment condition: (a) no treatment, (b) 100:0, (c) 0:100, (d) 50:50, (e) 70:30, (f) 90:10, (g) 30:70 liposomes:antibody, (h) cold antibody and (i) cold liposomes. On day 0, PC3-PIP tumor-bearing NSG male mice were treated with different ratios of tumor-responsive liposomes loaded with ^{225}Ac-DOTA and/or the PSMA-targeting ^{225}Ac-DOTA-SCN-antibody, at a total (single I.V.) dose equal to 4.63kBq per 20g animal.

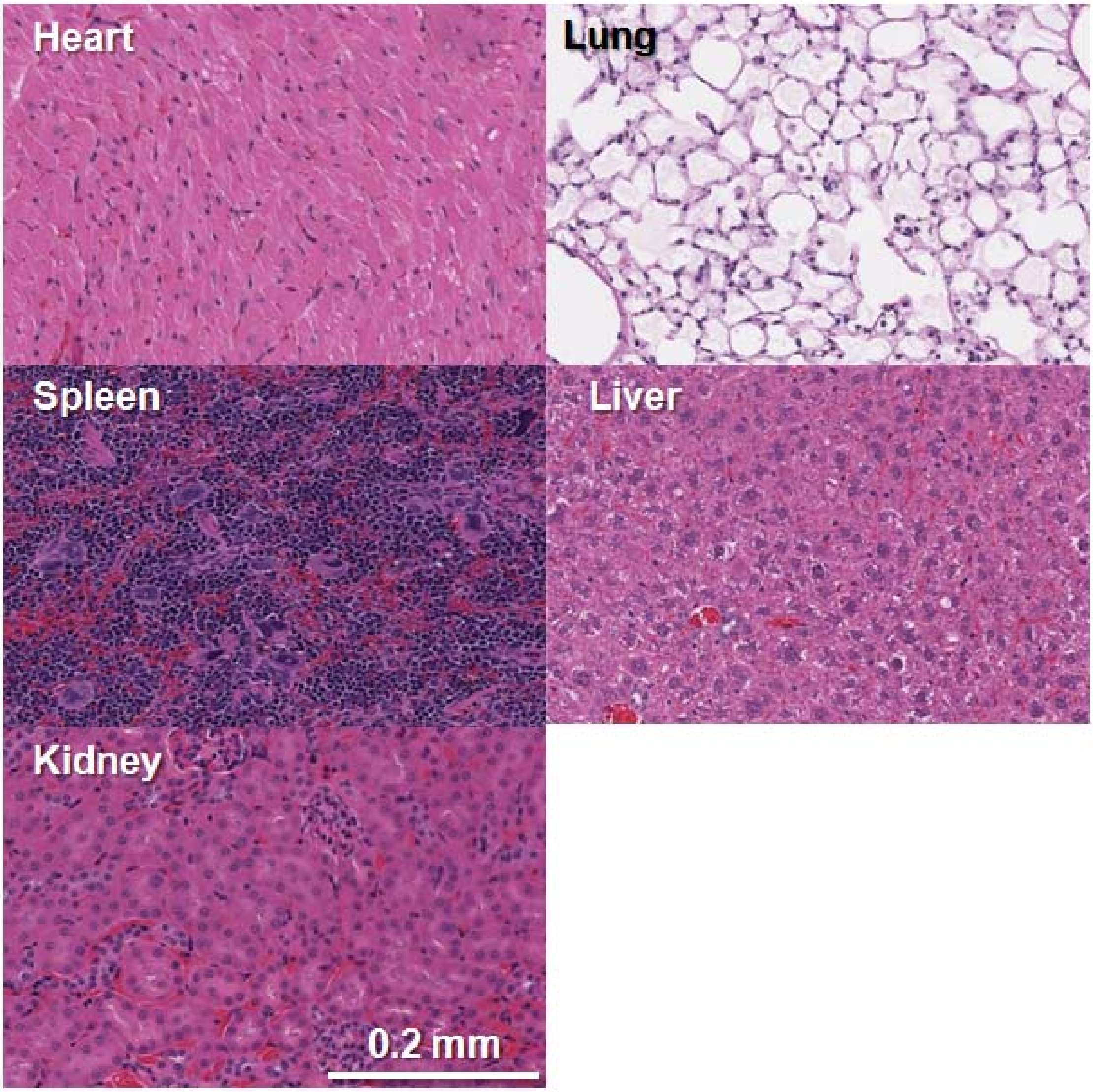

FIGURE S14 Histopathology evaluation from toxicity study using the PSMA-targeting antibody. No toxicities were observed on NSG mice one month after injection of the same total radioactivity that was used in the treatment study. Tumor-free NSG male mice were injected I.V. with 4.63 kBq ^{225}Ac, per 20 g animal, in the form of the PSMA-targeting ^{225}Ac-DOTA-SCN-antibody. The MTD of liposomal ^{225}Ac on tumor-free NSG mice was previously evaluated to be 5.55 kBq per 20 g animal [1].

References

[1] A. Prasad, R. Nair, O. Bhatavdekar, M. Sempkowski, A. Josefsson, J. Pancheco-Torres, Z.M. Bhujwalla, K. Gabrielson, G. Sgouros, S. Sofou, Transport-oriented engineering of liposomes for delivery of α-particle radiotherapy: inhibition of solid tumor progression and onset delay of spontaneous metastases European Journal of Nuclear Medicine and Molecular Imaging in press (2021).

[2] G. Sgouros, J.C. Roeske, M.R. McDevitt, S. Palm, B.J. Allen, D.R. Fisher, A.B. Brill, H. Song, R.W. Howell, G. Akabani, W.E. Bolch, R.F. Meredith, B.W. Wessels, P.B. Zanzonico, MIRD Pamphlet No. 22 (abridged): radiobiology and dosimetry of alpha-particle emitters for targeted radionuclide therapy, J Nucl Med 51(2) (2010) 311-28.

[3] H. Song, R.F. Hobbs, R. Vajravelu, D.L. Huso, C. Esaias, C. Apostolidis, A. Morgenstern, G. Sgouros, Radioimmunotherapy of breast cancer metastases with alpha-particle emitter 225Ac: comparing efficacy with ^{213}Bi and ^{90}Y, Cancer Res 69(23) (2009) 8941-8.

[4] M.R. McDevitt, D. Ma, L.T. Lai, J. Simon, P. Borchardt, R.K. Frank, K. Wu, V. Pellegrini, M.J. Curcio, M. Miederer, N.H. Bander, D.A. Scheinberg, Tumor therapy with targeted atomic nanogenerators, Science 294 (2001) 1537-1540.

[5] M.W. Saltzman, Engineering Principles for Drug Therapy, Oxford University Press 2001.
